# Nucleation and Growth of Bipyramidal Yb:LiYF_4_ Nanocrystals—Growing Up in a Hot Environment

**DOI:** 10.1021/acs.chemmater.3c00502

**Published:** 2023-07-03

**Authors:** Jence T. Mulder, Kellie Jenkinson, Stefano Toso, Mirko Prato, Wiel H. Evers, Sara Bals, Liberato Manna, Arjan J. Houtepen

**Affiliations:** † Optoelectronic Materials Section, Faculty of Applied Sciences, Delft University of Technology, Van der Maasweg 9, 2629HZ Delft, The Netherlands; ‡ Electron Microscopy for Materials Science (EMAT), Department of Physics, University of Antwerp, Groenenborgerlaan 171, 2020 Antwerp, Belgium; § Department of Nanochemistry, Istituto Italiano di Tecnologia (IIT), Via Morego 30, 16163 Genova, Italy; ∥ Materials Characterization Facility, Istituto Italiano di Tecnologia (IIT), Via Morego 30, 16163 Genova, Italy; # Department of Bionanoscience, Kavli Institute of Nanoscience, Delft University of Technology, van der Maasweg 9, 2629HZ Delft, The Netherlands

## Abstract

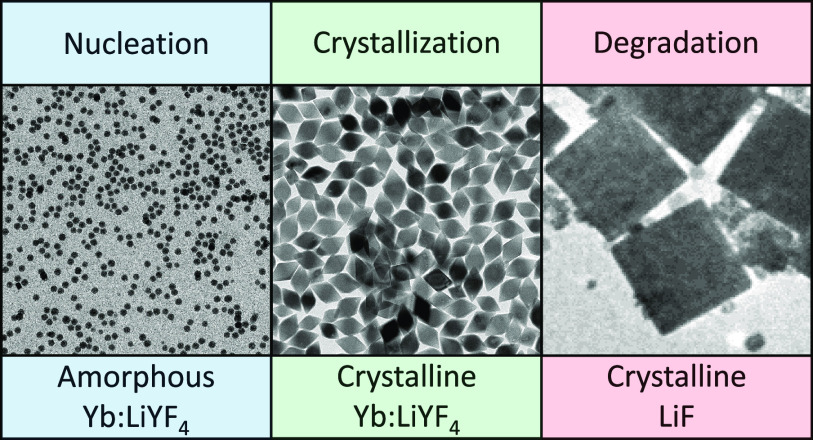

Lanthanide-doped LiYF_4_ (Ln:YLF) is commonly used for a broad variety of optical applications, such as lasing, photon upconversion and optical refrigeration. When synthesized as nanocrystals (NCs), this material is also of interest for biological applications and fundamental physical studies. Until now, it was unclear how Ln:YLF NCs grow from their ionic precursors into tetragonal NCs with a well-defined, bipyramidal shape and uniform dopant distribution. Here, we study the nucleation and growth of ytterbium-doped LiYF_4_ (Yb:YLF), as a template for general Ln:YLF NC syntheses. We show that the formation of bipyramidal Yb:YLF NCs is a multistep process starting with the formation of amorphous Yb:YLF spheres. Over time, these spheres grow via Ostwald ripening and crystallize, resulting in bipyramidal Yb:YLF NCs. We further show that prolonged heating of the NCs results in the degradation of the NCs, observed by the presence of large LiF cubes and small, irregular Yb:YLF NCs. Due to the similarity in chemical nature of all lanthanide ions our work sheds light on the formation stages of Ln:YLF NCs in general.

## Introduction

Lanthanide-doped nanocrystals (NCs) are an important class of optical materials, with applications that range from upconversion^1−3^ for *e.g*. medical studies,^4−6^ optical refrigeration for remote cooling,^7−11^ phosphors for lighting and display applications (especially in micro-LEDs where conventional phosphor particles are too large),^12,13^ to nanoscopic luminescent thermometers^14−17^ and nanoscintillators.^18−20^ The origin of their versatility in uses and properties lies in the existence of suitable host materials that can be synthesized as NCs and the broad variety of optical transitions, as well as a long excited state lifetime, of the elements in the lanthanide series.^21^

The host material that forms the basis of these NCs should be transparent and accommodate optically active lanthanide ions. For this, the use of yttrium-based hosts is ideal as Y^3+^ has a similar size and oxidation state as the elements in the lanthanide series. LiYF_4_ stands out because it fulfills all these conditions and can be prepared with a very high optical and crystal quality.^22,23^ An additional benefit over NaYF_4_, another common host material for lanthanide-doped NCs,^24−26^ is the more facile shell growth for LiYF_4_ as no phase changes^27,28^ occur. This furthermore allows subsequent growth of LiYF_4_ NCs to the preferred size by simply adding additional precursors.^23^ LiYF_4_ NCs thus form one of the most important platforms for optically active nanomaterials, and its synthesis would benefit from improved understanding of the nucleation and growth mechanisms leading to well-defined, monodisperse, and highly crystalline nanocrystals with a uniform distribution of the dopant ions.

Much work has been done on optimizing the synthesis of doped LiYF_4_ NCs through a variety of routes, often yielding NCs with a bipyramidal shape (*vide infra*).^23,29−37^ Little is known, however, about the complex nucleation and growth mechanisms of this material, with only a few works focusing on the nucleation stage.^32,34,35,38^ When ammonium fluoride is used as the fluoride precursor, instead, reports mention that LiF^34^ or YF_3_^22,39^ nucleate first. Reports using the thermal decomposition of trifluoroacetate (TFA) as the fluoride precursor show the initial formation of semispherical LiYF_4_ particles.^32,35^ None of these reports however discusses how these initial particles grow into a monodisperse ensemble of bipyramidal NCs. However, most works do not even show the initial synthesis stages and directly use complex, generally codoped NCs for optical studies. A better understanding of these important stages of NC formation may result in increased control of doped LiYF_4_ NC synthesis and improvement of the quality of the obtained NC samples. This, for example, increases the applicability of LiYF_4_ NCs for size- and shape-specific studies significantly.^40,41^

In this work, we focus on the nucleation and growth of ytterbium-doped LiYF_4_ (Yb:YLF), as most of the studied lanthanide-doped LiYF_4_ (Ln:YLF) NCs contain a significant fraction of Yb^3+^ either as the luminescent ion^22,23^ or as activator for other lanthanide ions.^22,42^ Yb:YLF itself is an interesting material for a broad range of optical applications. As bulk Yb:YLF, it is used in lasers,^43−45^ for photon upconversion (when codoped with other lanthanide ions such as Pr^3+^, Ho^3+^, Er^3+^, or Tm^3+^)^46−50^ and optical refrigeration.^11,51−53^ When synthesized as NCs, its applicability in biological^9,54−57^ and physical studies^11,22,40,41,58,59^ increases significantly. This becomes especially relevant when NC samples with a monodisperse size distribution^23,29,60^ and excellent optical properties (*e.g*. a high photoluminescence quantum yield^23,30^) are synthesized. Using Yb^3+^ additionally allows us to study the uniformity of its distribution and observe differences in the nucleation and growth of lanthanide-doped NCs, and hence if and how doping LiYF_4_ with lanthanide ions affects the formation of the NCs. The difference in ionic radii compared to Y^3+^ is in the lanthanide series one of the largest for Yb^3+^ (101.9 and 98.5 pm for the 8-coordinated ions respectively^61^); hence we expect that any conclusions we draw can be translated to LiYF_4_ NC syntheses when other, or additional, lanthanide ions are used as dopant. To verify this we have also synthesized Er:YLF NCs and find that their size, shape, and crystal structure are the same as for Yb:YLF NCs. This study thus acts as a general template for LiYF_4_ NC syntheses on which any more complex lanthanide-doped LiYF_4_ NC synthesis can be based.

We report the investigation of the nucleation and growth of Yb:YLF NCs using TFA precursors. We have followed the synthesis by taking aliquots of the synthesis mixture and analyzed the recovered materials using transmission electron microscopy (TEM), including high-angle annular dark-field scanning transmission electron microscopy (HAADF-STEM), electron diffraction (ED), and elemental analysis through energy dispersive X-ray spectroscopy (EDX). ED and X-ray diffraction (XRD) of the initially formed spherical particles did not show any clear signs of crystallinity. We identified that these initial particles are amorphous, formed as a product of rapid aggregation. In agreement with similar works on hydrothermal syntheses of Ln:YLF, we initially observe local short-ranged YF_3_ networks. However, in contrast to what was previously reported, we show that the particles already contain Li^+^ ions (approximately in a ratio expected for Yb:YLF). These particles thus do not consist of Yb:YF_3_ but of amorphous Yb:YLF, where the constituent ions are not yet arranged in the regular pattern of the LiYF_4_ crystal structure. Over time, the Yb:YLF spheres crystallize into bipyramidal NCs, the intended product. When the synthesis was extended for a day, large cube-shaped LiF crystals were recovered along with small, irregular Yb:YLF NCs. As along the LiF cubes no YF_3_ was found, or could be synthesized using only Y(TFA)_3_, we explain that the Yb:YLF NCs disintegrated due to dissolution of the material. As we furthermore show that LiF is much less soluble than YF_3_, we rationalize that LiF recrystallizes and that the yttrium ions are dissolved in the synthesis solvents. This suggest that the thermodynamic ground state of the system in these conditions is formed by LiF crystals and dissolved Y^3+^ and F^–^ ions. The LiYF_4_ NCs are a metastable intermediate that forms due to the more rapid kinetics of the nucleation. As the initially formed, amorphous Yb:YLF spheres can be isolated and purified before the crystallization is complete, we recommend further studies on amorphous spherical Yb:YLF particles as low-reactivity Yb:YLF precursors. These particles may, when undoped, especially be useful for *e.g*. novel shelling methods.

## Results

### Snapshots of an Yb:YLF Nanocrystal Synthesis

The synthesis of Yb:YLF NCs is carried out using a protocol we recently reported,^23^ which is based on the synthesis of Yi et al.^38^ with an additional precursor drying step from Homann et al.^62^ which we modified for the TFA-based synthesis method. The synthesis starts when anhydrous metal TFA salts (M^x+^(TFA)_*x*_, M = Li^+^, Y^3+^, Yb^3+^) are heated up in a high boiling point solvent (1-octadecene) with the addition of growth-controlling ligands (oleic acid). The TFA anion thermally decomposes between 267 °C^63^ and 310 °C^64^ releasing fluoride anions, as described in eq 1.
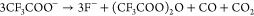
1

The temperature is increased stepwise (5 °C/30 s) to 330 °C, at which point all TFA is decomposed,^63^ and the solution is maintained for an additional 25 min at this temperature to allow the NCs to grow. After this, the synthesis mixture is cooled down to stop further growth, and subsequently the formed NCs are recovered.

The release of the fluoride anions results in the supersaturation of one or more fluoride compounds, hence inducing the nucleation of the fluoride based NCs. As Yb:YLF is a ternary compound and Yb^3+^, Y^3+^, Li^+^, and F^–^ ions are all present in the reaction mixture, it is not obvious which material, LiF, YF_3_, YbF_3_, or Yb:YLF, will form. Following the classical nucleation theory,^65,66^ the activation energy Δ*G* for the nucleation of a spherical particle, which is composed of a volume free energy term and a surface energy term, equals:
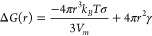
2where *r* = radius of the nucleus, *k*_*B*_ = Boltzmann constant, *T* = temperature, σ = supersaturation = , where μ_*ss*_ and μ_*eq*_ are the supersaturation and equilibrium chemical potentials, *V*_*m*_ = monomer volume, and γ = surface tension.

The activation energy for nucleation corresponds to the maximum in the curve given by eq 2 and is given by:
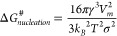
3From here, it is clear that at a fixed synthesis temperature the surface tension and the supersaturation (or, at fixed precursor concentration, the solubility) are the most important parameters in the nucleation rate of NCs and will determine which material (LiF, YF_3_, YbF_3_, or Yb:YLF) nucleates first.

**Figure 1:**
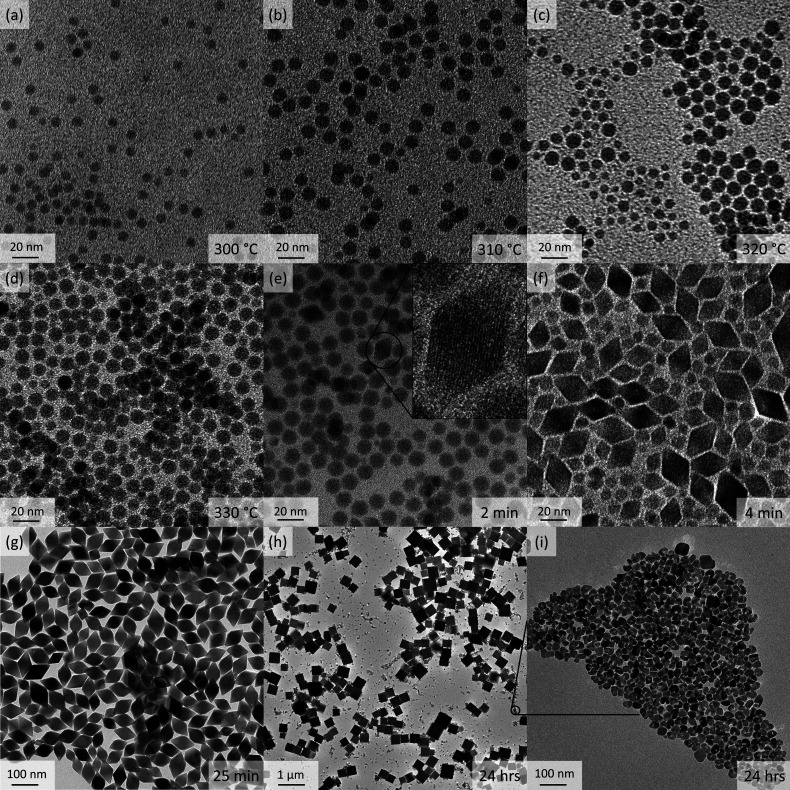
The nucleation and growth steps of Yb:YLF NCs. Aliquots taken during heating up at (a) 300 °C, (b) 310 °C, (c) 320 °C, and (d) 330 °C showcase the different stages of nucleation and growth of the Yb:YLF nanospheres. Aliquots taken during the NC growth at 330 °C, after (e) 2 min and (f) 4 min, highlight their transformation to crystalline NCs (see inset in (e)). (g) After 25 min, the synthesis mixture is cooled down and after purification the grown Yb:YLF NCs are recovered. (h) If the synthesis is continued at 330 °C for 24 h, large cubic crystals are found, together with (i) small particles.

To investigate the nucleation and growth of Yb:YLF NCs, a synthesis was followed by taking aliquots at different time steps, as shown in Figures 1 and S1. We first discuss the general observation from TEM imaging and then go into a more detailed analysis. Aliquots taken up to 290 °C did not yield any particles; thus it can be concluded that the concentration of the released fluoride ions through thermal decomposition is up to this point too low to initiate the nucleation of the NCs. When reaching 300 °C, many spherical NCs of roughly 7 nm in diameter are recovered, as shown in Figure 1a. This indicates that, at this temperature, lower than reported in some studies for Y(TFA)_3_,^64^ sufficient TFA-precursors have decomposed to initiate the nucleation. At 310 °C (Figure 1b) all NCs have grown, suggesting predominant growth from free precursors in solution, although the distribution splits in slightly larger and slightly smaller NCs (11.0 and 10.3 nm, respectively). At 320 °C (Figure 1c) a bimodal size distribution of spheres is clearly visible, where the smaller particles (7.0 nm) are smaller than those observed at 310 °C. This implies that the system has entered the Ostwald ripening regime, where larger particles grow through accretion from monomers that are being released from the smaller ones. Oswald ripening causes a broadening of the size distribution, but in its simplest treatment it should not lead to a bimodal distribution. However, in real-world scenarios where local interactions among dissolving/coarsening particles cannot be neglected, bimodal particle distributions have been described.^67^ Due to a large number of unknown parameters, we cannot pinpoint how or why this is the case for these nanospheres.

When reaching 330 °C, most of the small spheres have disappeared. After 2 min, the first crystalline, bipyramidal NCs appear (see inset Figure 1e). After 4 min, the majority of the NCs consist of crystalline bipyramids, although not yet with the narrowest size distribution. After 25 min (Figure 1g) an NC sample clear of any spherical particles and with a narrow size distribution is obtained. If the solution is kept at 330 °C for 24 h, almost micrometer-sized, cube-shaped crystals are obtained together with a large amount of small, irregularly shaped but crystalline particles, as shown in Figure 1h,i.

These observations raise a number of questions. First of all, it is yet unclear what the small spheres that nucleate first are composed of. Understanding their composition is key, as these particles form the basis from which the Yb:YLF NCs grow. Second, is it not straightforward how spheres transform into strongly faceted Yb:YLF NCs. And third, a clarification is required on how bipyramidal Yb:YLF NCs finally transform into cube-shaped crystals. Below, we investigate the various synthesis stages in more detail to answer these questions.

### Analysis of the Initial Nanospheres

**Figure 2:**
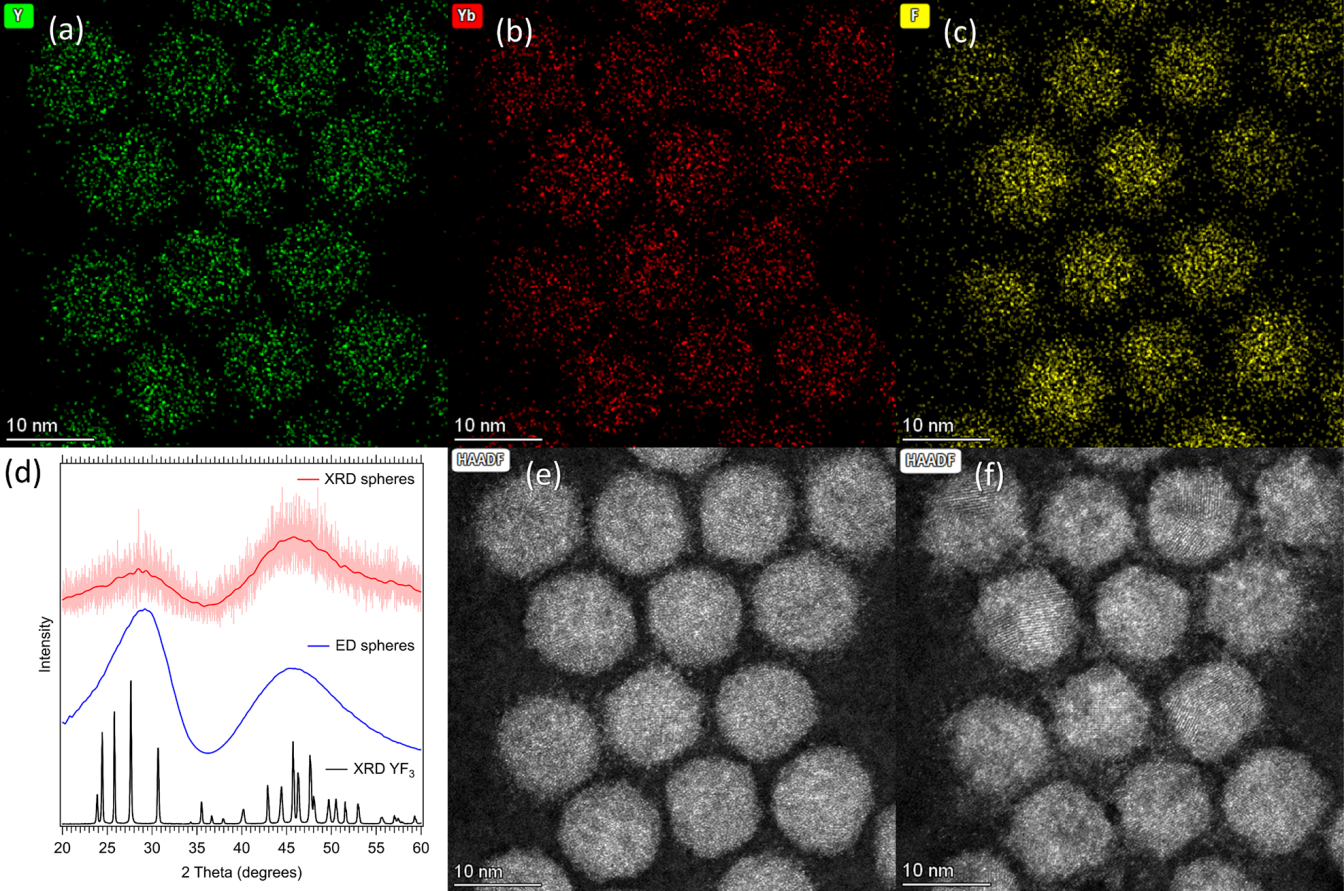
Analysis of the initially nucleated nanospheres. (a) EDX measurements showing the presence and location of yttrium, (b) ytterbium, and (c) fluoride ions in the Yb:YLF nanospheres. (d) ED and XRD both show no signs of sharp reflections, indicating that the material is mostly amorphous. There is a similar structure in both sphere measurements, which overlaps well with an XRD of YF_3_. This indicates that there is short-range Y–F order in the spheres; however, the particles are still mostly amorphous. (e) The first and (f) twelfth frames of an HAADF-STEM measurement, showing the crystallization of the initially amorphous nanospheres (e) as a result of electron beam exposure. From the crystallized particles (f), the lattice spacing of Yb:YLF was obtained.

HAADF-STEM images of the first sample of spheres, recovered at 300 °C, are shown in Figures 2 and S2. From EDX measurements (Figure 2a–c) it is clear that the spheres contain yttrium, ytterbium, and fluoride ions with a uniform Y:Yb distribution (SI-3). Lithium, however, is too light to be measured with EDX (atomic number Z = 3). The spheres can thus either consist of Yb:YF_3_ or Yb:YLF. To better understand the composition of the spheres, ED and XRD were performed (on a separately synthesized scaled-up batch of spheres), as shown in Figure 2d. The diffractograms do not show any sharp diffraction peaks, even though the NCs are large enough to give defined diffraction signals. As both methods give the same diffraction pattern, which overlaps best with an XRD of YF_3_, we conclude that the particles do have short-range YF_3_-like order. In agreement with this conclusion, XRD patterns simulated through the application of the Debye scattering equation to isotropic crystalline domains of YF_3_ about 1.5–2 unit cells in size show strong similarities with the experimental data, while similar simulations performed for LiYF_4_ do not provide a reasonable match (SI-5). Together with the first frame of the HAADF-STEM image (Figure 2e), it can be concluded that the spheres do not show signs of high-order crystallinity and are thus mostly amorphous (see also SI-6). This is also in line with photoluminescence measurements (SI-7), which show a single sharp emission peak at 976 nm. If the material is crystalline Yb:YLF, three emission peaks, the signatures of crystal field splitting on the Yb^3+^ transitions, are expected (SI-7). Instead, the emission spectrum is similar to that of free Yb^3+^ ions,^68^ confirming that the Yb^3+^ ions are not yet incorporated in a crystalline LiYF_4_ host configuration.

We can however conclude that the nanospheres are composed of amorphous Yb:YLF, as in a later frame of the HAADF-STEM measurement (Figure 2f and SI-8) clear crystal planes are visible. This indicates that the spheres partially crystallized under the electron beam (acceleration voltage of 300 kV). After crystallization, the lattice spacing corresponding to the (112) plane of LiYF_4_ is observed (SI-8, SI-9, and SI-10). X-ray photoelectron spectroscopy (XPS) measurements furthermore confirm the presence of Li^+^ ions (Li:Y/Yb:F = 1:1.5:5.4) (SI-11). Additionally, thermal annealing of the spheres (which were washed thrice, in order to remove any unincorporated ions) results in the formation of crystalline material, as shown in Figure S13c. In agreement with the XPS results, the XRD of the annealed spheres shows a strong diffraction signal corresponding to Yb:YLF, with the presence of a minor fraction of YF_3_. From the obtained lattice spacing, XPS, and XRD after annealing we conclude that the original nanospheres consist of amorphous Yb:YLF.

We explain the formation of amorphous Yb:YLF as a result of a rapid nucleation of Yb:YLF once fluoride ions are released, capturing the ions in the location where they arrive without annealing into a crystalline structure.

HAADF-STEM measurements on a sample isolated at 320 °C, similar to Figure 1c, show no difference between the smaller and larger spheres in the bimodal size distribution (see Figure S14). Therefore, we conclude that both sizes consist of amorphous Yb:YLF; hence no second material has nucleated. To answer why the different sizes of spheres are found, we performed a size analysis on the TEM images that are shown in Figure 1a–e.

### Growth via Ostwald-Ripening and Metamorphosis into Bipyramidal Nanocrystals

**Figure 3:**
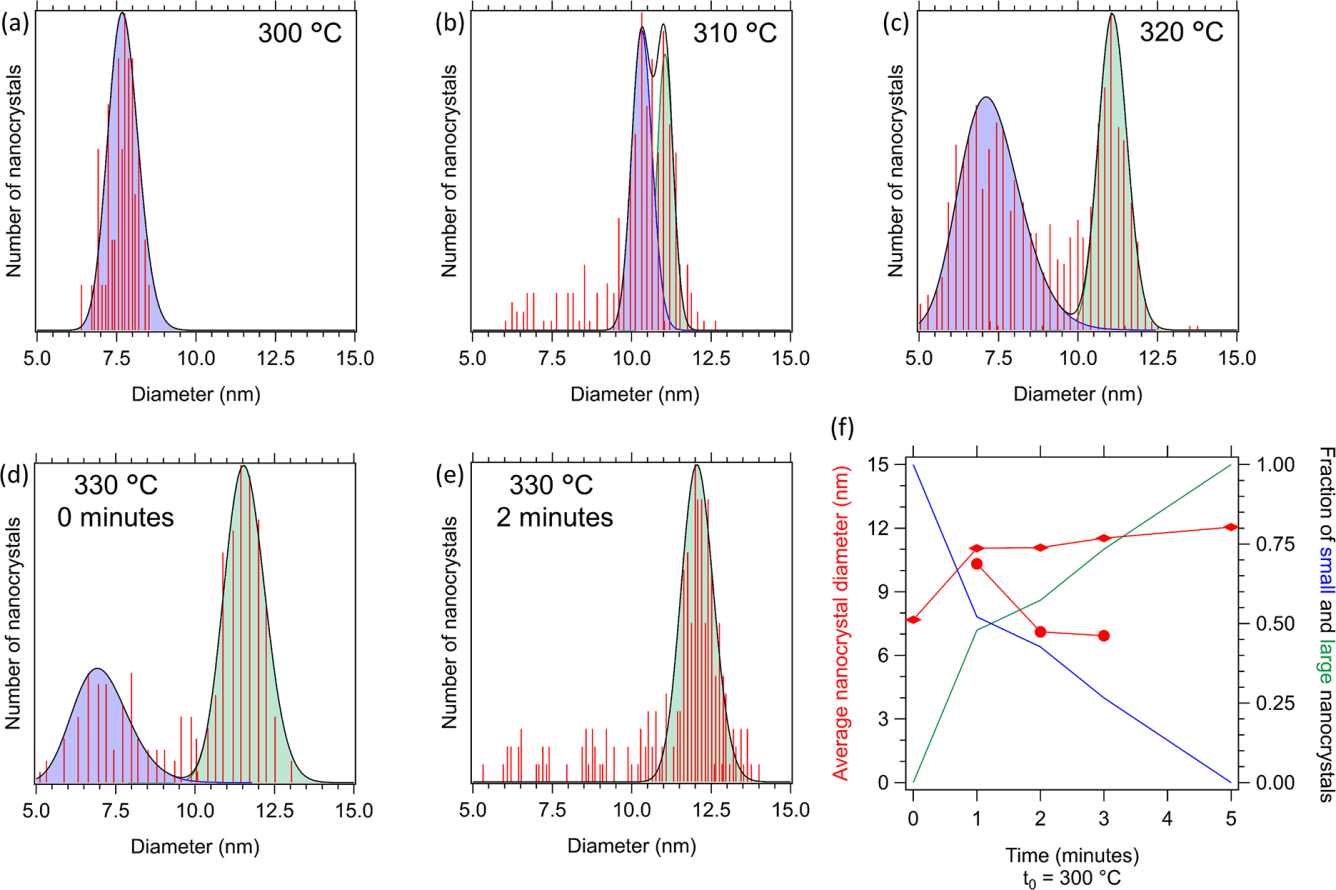
(a–e) Size distributions of NCs based on the TEM images shown in Figure 1a–e. All fits are log-normal plots, used to determine the average size and sphere ratios shown in (f).

Figure 3 shows the size distributions of the first growth stages by fitting a log-normal distribution to the distribution of measured diameters obtained from the TEM images. From here, we observe that initially all nanospheres grow equally (Figure 3a). This indicates that volume is added to all NCs and that, therefore, growth takes place via the addition of monomers from a supersaturated solution. Subsequently, a bimodal distribution of diameters is observed, where roughly half of the spheres reduce in size and half of the spheres grow (Figure 3b). Over time, the smaller nanospheres, which are abundant at first (Figures 3c and S15), reduce in number until solely the larger spheres are left (Figures 3d,e). This clearly shows that growth becomes dominated by the dissolution of the smaller particles. Most likely the concentration of free monomers is depleted to the equilibrium concentration of the Yb:YLF spheres after a temperature of 310 °C is reached and further growth can only take place via Ostwald ripening. Around the same time that the small spheres are practically gone, the first crystalline Yb:YLF NCs appear. As the volume of the large spheres and the initial Yb:YLF NCs are similar (SI-14), we conclude that the YF_3_ structures have thermodynamically rearranged to form crystalline Yb:YLF NCs. Over time, all spheres disappear and the tetragonal Yb:YLF NCs increase in concentration and size.

### From Bipyramidal to Cubic Crystals

**Figure 4:**
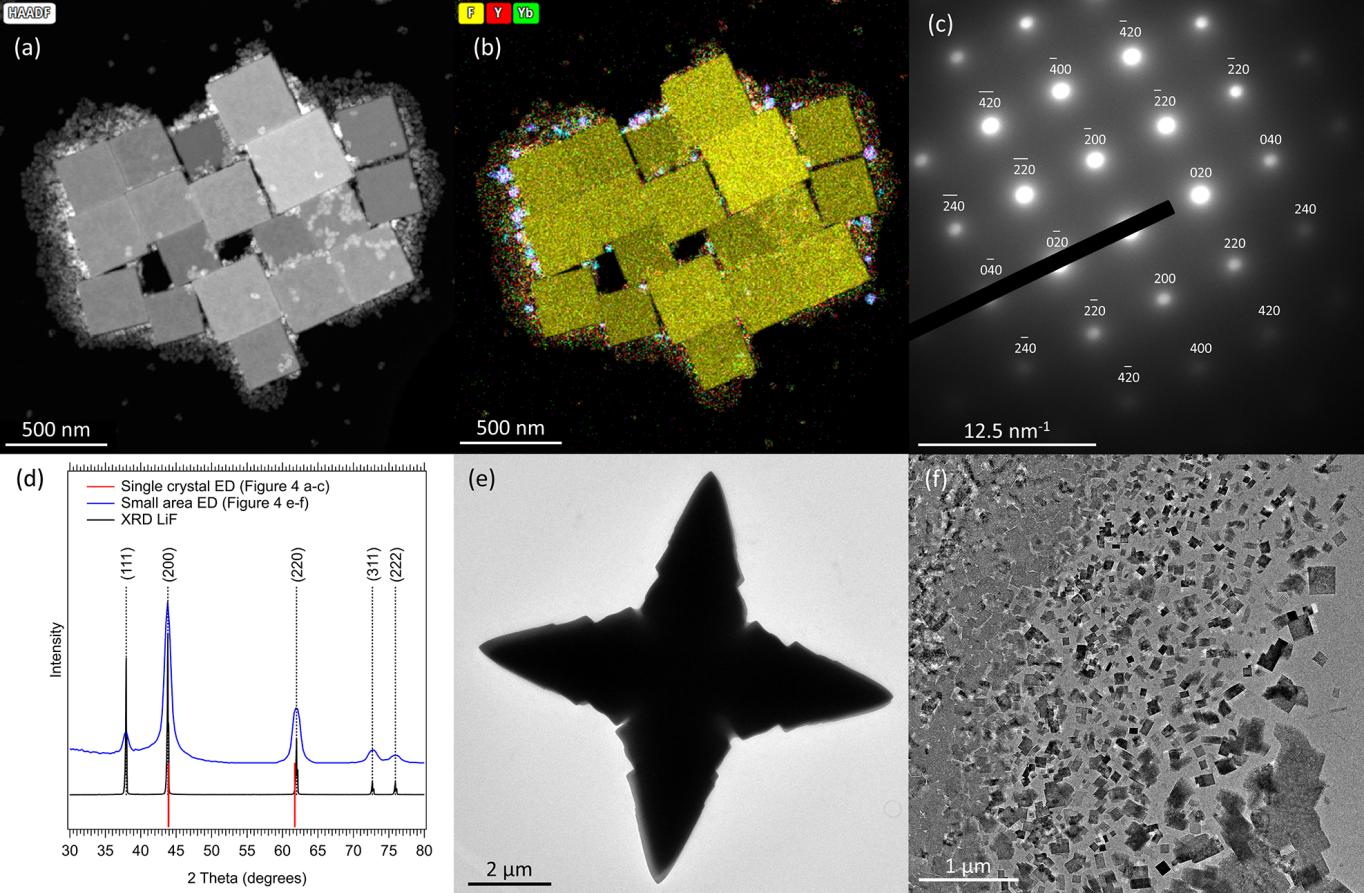
Analysis of the micrometer-sized cubes. (a) HAADF-STEM and (b) EDX measurements performed on the large cubic crystals, showcasing the absence of yttrium and ytterbium in the material. (c) Single crystal ED shows that the material has a face centered cubic crystal structure. The analysis of this ED, shown for the (200) and (220) reflections in (d), confirms that the cubes are composed of LiF. (e) Performing a synthesis identical to the Yb:YLF NC synthesis, but with solely LiTFA, yields large, shuriken-shaped LiF crystals. The crystal shown here was recovered during heating up when reaching 300 °C, the temperature where otherwise the small amorphous Yb:YLF nanospheres are recovered. (f) The shuriken-shaped crystals disintegrated swiftly under the electron beam, resulting in the formation of small cubic particles. The small area ED of these particles, shown in (d), confirms that this is, as expected, LiF.

When keeping the synthesis mixture at 330 °C for 24 h instead of the usual 25 min, cube-shaped, almost micrometer-sized crystals were found (shown in Figure 4a). EDX measurements (Figure 4b) showed no presence of yttrium or ytterbium ions, but a strong fluoride signal was recorded. The analysis of a single crystal ED (Figure 4c,d) indicated that the crystal has a cubic crystal structure, for which the reflections correspond with those for LiF. Therefore, either (1) LiF crystals nucleated at longer time scales, from residual free Li^+^ and F^–^ ions, or (2) the Yb:YLF NCs dissolved and the freed monomers recrystallized, forming LiF. As apart from small, irregularly shaped Yb:YLF NCs with a large surface area (SI-15 and SI-16) no bipyramidal Yb:YLF NCs (as shown in Figure 1g) were found, the LiF is most likely a product of the decomposition of the Yb:YLF NCs. The irregularly shaped Yb:YLF NCs are in that case remaining fragments of partially dissolved Yb:YLF NCs. This is furthermore in line with the observed shapes of the fragments, which have a strong resemblance with (parts of) the original bipyramidal NCs. Remarkably, no Yb:YF_3_ was found, which would be the expected leftover material after removing LiF from Yb:YLF. This suggests that Yb:YF_3_ is soluble in the synthesis mixture without the presence of free Li^+^. To gain more insight, we attempted to synthesize LiF by performing an identical Yb:YLF NC synthesis (keeping [TFA] the same), but then by using only LiTFA. Starting at 285 °C, which is earlier than the first observed nuclei during the Yb:YLF NC synthesis, very large and out of equilibrium LiF crystals (*i.e*. with a large surface over volume ratio), resembling shurikens, were obtained (as shown in Figure 4e and Figure S19). This shows that the nucleation of LiF is slow, hence only few nuclei form which can then grow very large from the initial growth from solution. These crystals were swiftly affected by the electron beam, disintegrating into cubic LiF crystals (Figure 4f). These observations are all in line with a much higher activation barrier for LiF nucleation than that of Yb:YLF, determined by the surface tension γ; hence LiF nuclei are not observed in a regular Yb:YLF NC synthesis.

### Proposed Nucleation and Degradation Model and Discussion

**Figure 5:**
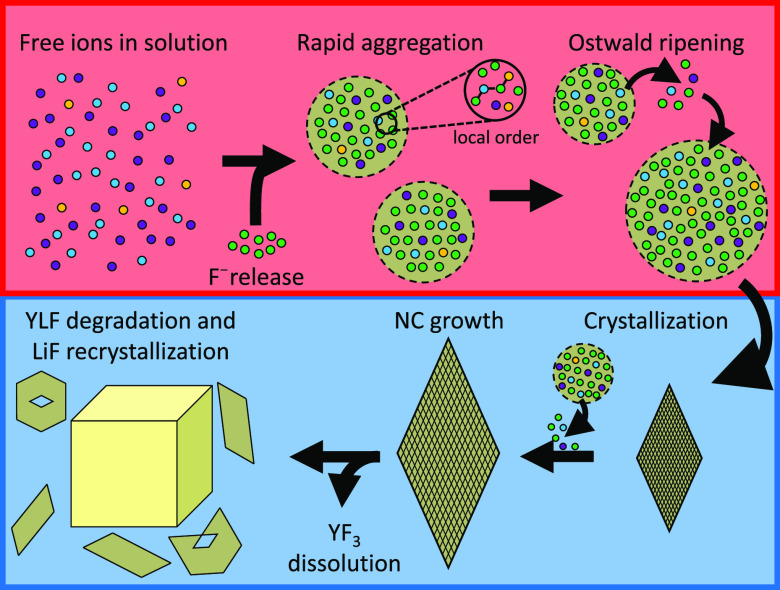
Schematic of the proposed nucleation and growth mechanism. The nanocrystal synthesis starts from the release of fluoride ions through thermal decomposition of the TFA precursor, followed by a rapid aggregation of all ions, resulting in amorphous Yb:YLF spheres with local YF_3_ order. Once the fluoride concentration in the solution lowers, Ostwald ripening is observed. Subsequently, the largest spheres crystallize in bipyramidal Yb:YLF NCs which can be recovered, or grown, until the solution is depleted from spheres. If the synthesis mixture is kept at the synthesis conditions for 24 h, Yb:YLF NCs degrade through slow dissolution. The freed lithium and fluoride ions recrystallize as large LiF cubes.

The observations and analyses we discussed so far are summarized in Figure 5. In short, all the cations that are free in solution (*i.e*. Li^+^, Y^3+^, and Yb^3+^) rapidly aggregate once sufficient F^–^ ions are released due to thermal decomposition of the TFA anion. The aggregation is random, and faster than the crystallization, so that all ions are incorporated into the amorphous spheres at the location where they arrive. Although ED and XRD show the presence of short-range YF_3_ order, XPS reveals that the spheres contain a large fraction of lithium ions (Li:Y/Yb:F = 1:1.5:5.4). As, moreover, thermal annealing of the spheres shows that this material crystallizes as Yb:YLF, we conclude that the spheres consist of amorphous Yb:YLF. Over time, the spheres grow via Ostwald ripening, until the ions are incorporated into the LiYF_4_ lattice positions (*i.e*. crystallization). At this moment, crystalline bipyramidal Yb:YLF NCs are formed. These NCs continue to grow due to the supply of ions from the amorphous spheres, until all spheres have either crystallized or dissolved. If, however, the temperature is not lowered, but the synthesis mixture is kept at 330 °C for 1 day, large LiF cubes and residual Yb:YLF fragments are observed. We conclude that this is caused by the slow dissolution of the bipyramidal Yb:YLF NCs and subsequent recrystallization of the product with the lowest solubility: LiF.

The proposed model leaves room to speculate what happens to the remaining Y^3+^, Yb^3+^, and F^–^ ions. To understand this, we performed multiple attempts to synthesize YF_3_ NCs in a similar manner as the LiF, by using solely Y(TFA)_3_. This repeatedly did not yield any particles; hence we conclude that the solubility of YF_3_ is too high under the synthesis conditions to initiate nucleation in the absence of Li^+^ ions. This also explains why no YF_3_ crystals were found after the decomposition of LiYF_4_ into LiF: the freed Y^3+^ and F^–^ ions are simply dissolved, following eq 4.
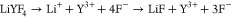
4

The rapid initial aggregation of all ions, resulting in the formation of amorphous Yb:YLF spheres, is thus driven by the presence of Li^+^ ions, and key in the formation of Yb:YLF. We conclude that LiF has the lowest solubility; hence this material corresponds to the thermodynamic ground state under the synthesis conditions. However, due to the much faster nucleation of Yb:YLF this material forms first, and only prolonged heating induces the conversion to LiF. Another positive consequence of the rapid aggregation is the uniform distribution of Y^3+^ and Yb^3+^ in the spheres, which is especially important for multiphoton upconversion. This, as energy transfer is required from at least two excited Yb^3+^ ions to one codopant (*e.g*. Pr^3+^, Ho^3+^, Er^3+^, or Tm^3+^) and as we have shown before,^23^ energy transfer has a steep distance dependency.

In the Introduction we mentioned that, due to the chemical similarities of all lanthanide ions, we expect that this work can be translated to LiYF_4_ NC syntheses with other dopants than Yb^3+^. To test this, we synthesized 30% erbium-doped LiYF_4_ NCs. The resulting sample has the same NC size and shape, crystal structure, and expected Er^3+^ doping fraction, and additionally shows PL that is specific to Er^3+^,^69^ as is shown in Supporting Information SI-19. This shows that the nucleation and growth of Er:YLF and Yb:YLF proceed in a similar way and suggests that it may be general for lanthanide-doped YLF NCs.

The facile synthesis of the amorphous (Yb:)YLF nanospheres may be interesting for other uses. As these particles can easily be purified, they can be used as a much less reactive Yb:YLF precursor in comparison with the TFA-based precursors. Beneficially, they can be applied at any temperature independent of the F^–^ release from the TFA precursors, mitigating the possibility of ion migration from the doped core to the undoped shell as reported before.^70,71^ In a somewhat analogous manner, for hexagonal NaYF_4_ NC shelling methods, small cubic NaYF_4_ NCs are used as a sacrificial shell growth precursor for core–shell NaYF_4_@Ln:NaYF_4_.^24,25,62,72,73^ We recommend further research in using these amorphous LiYF_4_ nanospheres as LiYF_4_ precursors, especially for lower-temperature syntheses and shelling methods.

## Conclusion

In this work, we followed an Yb:YLF NC synthesis using aliquots and electron microscopy techniques. We concluded that the formation of bipyramidal Yb:YLF NCs by using the thermal decomposition of TFA precursors is a multistep process, which starts from the formation of amorphous Yb:YLF spheres. These spheres grow via Ostwald ripening, until thermodynamic rearrangement results in the formation of crystalline Yb:YLF NCs. Additional experiments show that if the synthesis mixture is kept at the synthesis conditions for 24 h, the Yb:YLF NCs degrade by dissolution. This results in the recrystallization of highly insoluble LiF crystals, whereas the remaining Y^3+^ and F^–^ ions remain in solution. From this, we inferred that Yb:YLF is a metastable intermediate that forms due to more rapid kinetics of the nucleation.

## Experimental Methods

### Materials

1-Octadecene (ODE, technical grade, 90%) and lithium fluoride (LiF, 99.995%, precipitated) were purchased from Sigma-Aldrich. Oleic acid (extra pure), trifluoroacetic acid (HTFA, ≥99.0%, for HPLC), trifluoroacetic anhydride (TFAA, ≥99.0%), lithium carbonate (Li_2_CO_3_, 99.999%, trace metal basis), yttrium oxide (Y_2_O_3_, 99.9999%, REO), erbium oxide (Er_2_O_3_, 99.9%, REO), and ytterbium oxide (Yb_2_O_3_, 99.998%, REO) were purchased from Fisher Scientific. Toluene (≥99.8%, anhydrous), methanol (anhydrous, max 0.003% H_2_O), and yttrium fluoride (YF_3_, 99.9%, anhydrous) were purchased from VWR Chemicals.

All chemicals were used as received, unless specified differently. All manipulations were performed under N_2_ atmosphere, using standard Schlenk line techniques or a nitrogen-filled glovebox (<0.1 ppm of H_2_O; <0.1 ppm of O_2_), unless otherwise mentioned.

Milli-Q water was obtained from a Milli-Q Advantage A10 system (Merck Millipore, 18.2 MΩ·cm, 2 ppb TOC).

### Synthesis and Drying of Metal Trifluoroacetate Precursors

Metal trifluoroacetate salts, M^*x*+^(TFA)_*x*_ (M = Li^+^, Y^3+^, Er^3+^, Yb^3+^), were synthesized by adding 5 mmol of Li_2_CO_3_ (369 mg), Y_2_O_3_ (1129 mg), Er_2_O_3_ (1913 mg), or Yb_2_O_3_ (1970 mg) and 5 mL of Milli-Q water to a 25 mL 2-necked flask with a fused thermocouple insert containing a PTFE-coated stirring bar. The flask was connected to a Schlenk line equipped with a water-cooled condenser, and 5 mL of trifluoroacetic acid (HTFA; ∼ 65 mmol) was added dropwise under stirring. *Note: as the reaction between HTFA and Li*_2_*CO*_3_*is strongly exothermic and results in the release of a large volume of CO*_2_, *the acid should be added carefully to avoid splashing*. After all of the acid had been added, the necks of the flask were closed with septa, and the reaction mixture was placed under a N_2_ gas flow. The mixture was heated to 120 °C and left under reflux until a clear, colorless solution was obtained (generally <1 min for Li_2_CO_3_, >1 h for Y_2_O_3_, Er_2_O_3_, and Yb_2_O_3_). At this point, the reaction mixture was cooled to below 50 °C, and a vacuum was applied to evaporate all water and acid. The resulting solids were transferred to a glovebox and crushed to form a white (or in the case of Er(TFA)_3_, pink) powder. These powders may appear dry, but are very likely to contain crystal water, especially in the case of lithium trifluoroacetate, which is extremely hygroscopic.

To remove the undesired crystal water, the precursor salts were dried further using trifluoroacetic anhydride (TFAA). Briefly, the powdered metal TFA salts were added to a 250 mL round-bottom flask, connected to a Schlenk line, and placed under nitrogen flow. TFAA (5 mL, ∼36 mmol) was added, resulting in a rise in the temperature of the reaction mixture. This mixture was left to stir for 1 h, after which the TFAA and HTFA were removed under vacuum. The remaining solids were transferred back to a nitrogen-filled glovebox and crushed to a powder. *Note: TFAA reacts aggressively with water and can damage plastic and rubber tubing. To protect the Schlenk line tubing and the vacuum pump, the evaporated TFAA was collected in an additional cold trap which was placed directly after the reaction flask. The cold trap contents were carefully quenched with isopropanol before disposal*.

### Following the Synthesis of Yb:YLF NCs

Yb:YLF NCs were synthesized according to a protocol we recently reported.^23^ To a two-necked round-bottom flask (25 mL) with fused thermocouple insert, 2 mmol of LiTFA (240 mg) and 2 mmol of a mixture of Y(TFA)_3_ (428 mg/mmol) and Yb(TFA)_3_ (515 mg/mmol) in the ratios required were added inside a nitrogen filled glovebox. To this, a mixture of 6.5 mL of ODE and 6.5 mL of OA, both previously degassed, was added. The flask was then attached to a Schlenk line without exposure to the air, and the contents were degassed at 100 °C for 1 h. Thereafter, the flask was put under a flow of N_2_, and the temperature was increased stepwise to 330 °C with increments of 5 °C/30 s. Aliquots of 200 μL were taken after every 10 °C and injected in 2 mL of toluene to monitor the NC nucleation and growth. Once the contents reached 330 °C, aliquots were taken after 2, 4, and 25 min. The reaction was left at 330 °C for 24 h and subsequently cooled down to room temperature using compressed air. The resulting mixture was yellow and opaque. Using a syringe, 2 mL of toluene was added to facilitate transfer from the flask to a nitrogen-filled vial. Methanol (∼5 mL) was added to the final synthesis mixture. The mixture was centrifuged at a relative centrifugal force of 1800 × g (3800 rpm) for 10 min, the supernatant was discarded, and the solid NC pellet was redispersed in 2 mL of toluene. To this, 1 mL of methanol was added, and after centrifugation, the NC pellet was redispersed in 2 mL of toluene. This step was repeated once more, and the final NC sample, dispersed in 2 mL of toluene, was stored in air. The aliquots were washed in a similar manner; however solely 0.5 mL of methanol was used per washing step, and the samples were redispersed in 1 mL of toluene each time.

### Synthesis of Yb:YLF Nanospheres

For the XRD measurement, shown in Figure 2d, a similar synthesis was carried out as explained above; however, once the synthesis mixture reached 300 °C, it was directly cooled down using compressed air. The nanospheres were isolated in an identical manner as described above for the final synthesis mixture; however, 20 mL of methanol was used for the first washing step in order to obtain as much product as possible. The final product, obtained after three washing steps, was again stored in 2 mL of toluene.

### Synthesis of Shuriken-Shaped LiF Crystals

Large LiF crystals were synthesized following the same procedure as described for the regular Yb:YLF NC synthesis, but by solely using 8 mmol of LiTFA (960 mg). An amount of 8 mmol was chosen in order to keep the total amount of released fluoride ions the same. Aliquots of 1 mL were taken at 270 °C, 285 °C, 300 °C, 315 °C, and 330 °C and added to 2 mL of toluene. After 25 min at 330 °C, the remaining synthesis mixture was washed following the washing steps described above, using 5 mL of methanol for the first washing step of the synthesis mixture and 1 mL of methanol for the aliquots. The final samples were all stored in 1 mL of toluene.

### Attempted Synthesis of YF_3_ Crystals

Identical to the synthesis of the shuriken-shaped LiF crystals mentioned above, we attempted to synthesize YF_3_ using 8/3 mmol Y(TFA)_3_ (1141 mg).

### Synthesis of Er:YLF NCs

Er:YLF NCs were synthesized according to the protocol we recently reported for the synthesis of Yb:YLF NCs, but instead of using Yb(TFA)_3_, Er(TFA)_3_ was used.^23^

### Optical Characterization

Emission spectra were obtained using an Edinburgh Instruments FLS980 spectrometer equipped with a liquid nitrogen-cooled NIR PMT-based detector from Hamamatsu. A Xenon arc lamp from XBO was used as an excitation source.

TRPL spectra were also obtained using an Edinburgh Instruments FLS980 spectrometer equipped with a liquid nitrogen cooled NIR PMT-based detector from Hamamatsu. The measurements were performed using time-correlated single photon counting, with 930 nm nanosecond laser pulses from a M8903-01 Hamamatsu laser unit as an excitation source.

### Structural Characterization

TEM and ED images were acquired using a JEOL JEM1400 transmission electron microscope operating at 120 kV, equipped with an SSD-EDX detector for spot (>75 nm) analysis. ED analysis was performed using CrysTbox^74^ software.

High-angle annular dark field scanning transmission electron microscopy images and energy-dispersive X-ray spectral maps were acquired using an aberration-corrected cubed Thermo Fisher Scientific-Titan electron microscope operated at an acceleration voltage of 300 kV equipped with a Super-X detector.

XRD measurements were performed with a Bruckner D8 ADVANCE diffractometer (Cu Kα, λ = 0.15406 nm). The NC samples were drop-casted on zero diffraction (911) silicon substrates unless stated differently. XRD simulations were performed using the Diffraction Analysis for Nanopowders (DIANNA) software,^75^ which implements the Debye scattering equation for the simulation of finite-size atomistic models of nanoparticles.

The XPS analyses were performed with a Kratos Axis Ultra^DLD^ spectrometer using a monochromatic Al Kα source (20 mA, 15 kV). Survey scan analyses were performed with an analysis area of 300 × 700 μm^2^ and a pass energy of 160 eV, whereas high-resolution analyses were performed with a pass energy of 20 eV. Specimens for XPS measurements were prepared by dropping a concentrated NC solution onto a freshly cleaved highly oriented pyrolytic graphite substrate (HOPG, ZYB).

## Supporting Information Available

Overview of the nucleation, growth, and degradation of Yb:YLF NCs, additional HAADF-STEM and EDX mapping of nanospheres and small particles, yttrium to ytterbium distribution in the nanospheres, XRD references of LiF, YF_3_, and LiYF_4_, electron diffraction analyses, simulation of unit cell reflections for ED and XRD, optical properties of Yb:YLF nanospheres, e-beam crystallization image frames, distinguishing between YF_3_ and LiYF_4_ from the location of the yttrium ions by HAADF-STEM, lattice parameter analysis of crystallized spheres, XPS analysis of Yb:YLF nanospheres, XRD on thermally annealed nanospheres, HAADF-STEM and EDX mapping of bimodal spheres and irregular YLF crystals, calculation of nanocrystal volumes, additional HAADF-STEM images of degraded YLF NCs, growth of shuriken-shaped LiF crystals, tomography of LiF cubes, and Er:YLF NC structural, compositional and optical properties (PDF)
