# Supplementary material for: Nucleation and Growth of Bipyramidal Yb:LiYF4 Nanocrystals—Growing Up in a Hot Environment
Source: Chem Mater. 2023 Jul 3;35(14):5311–21. doi: 10.1021/acs.chemmater.3c00502 (PMC10389792; doi:10.1021/acs.chemmater.3c00502)
Supplement: Supplementary file 1 — cm3c00502_si_001.pdf [file cm3c00502_si_001.pdf]

# Supporting information for

# Nucleation and Growth of Bipyramidal Yb:$LiYF_4$ Nanocrystals – Growing Up in a Hot Environment

*Jence T. Mulder[†], Kellie Jenkinson[‡], Stefano Toso[§], Mirko Prato[&], Wiel H. Evers[†,#], Sara Bals[‡], Liberato Manna[§], Arjan J. Houtepen[†]**

[†] Optoelectronic Materials Section, Faculty of Applied Sciences, Delft University of Technology, Van der Maasweg 9, 2629HZ Delft, The Netherlands

[‡] Electron Microscopy for Materials Science (EMAT), Department of Physics, University of Antwerp, Groenenborgerlaan 171, 2020 Antwerp, Belgium

[§] Department of Nanochemistry, Istituto Italiano di Tecnologia (IIT), Via Morego 30, 16163 Genova, Italy

[&] Materials Characterization Facility, Istituto Italiano di Tecnologia (IIT), Via Morego 30, 16163 Genova, Italy

[#] Department of Bionanoscience, Kavli Institute of Nanoscience, Delft University of Technology, van der Maasweg 9, 2629 HZ Delft, The Netherlands

**Address correspondence to A.J.Houtepen@tudelft.nl*

# Contents

SI-1 – Nucleation, Growth and Degradation of Yb:YLF Nanocrystals................................ S-1
SI-2 – HAADF-STEM and EDX Images of Yb:YLF Spheres............................................. S-2
SI-3 – Yttrium and Ytterbium Distribution in Yb:YLF Spheres .......................................... S-3
SI-4 – XRD References for LiF, $YF_3$ and $LiYF_4$................................................................. S-4
SI-5 – Structural Analysis of Yb:YLF Spheres and Simulations of Unit Cell Reflections ... S-5
SI-6 – Nanocrystal Analysis Using Electron Diffraction ..................................................... S-6
SI-7 – Optical Properties of Yb:YLF Nanospheres ............................................................. S-7
SI-8 – Analysis of Nanosphere Material Composition Through e-Beam Crystallization ..... S-8
SI-9 – Using the Location of Yttrium Ions to Distinguish Between $YF_3$ and $LiYF_4$ ............ S-9
SI-10 – Analysis of Nanosphere Composition Using its Lattice Parameter........................ S-10
SI-11 – XPS Measurements of Yb:YLF Nanospheres ....................................................... S-11
SI-12 – XRD on Thermally Annealed Nanospheres.......................................................... S-12
SI-13 – HAADF-STEM and EDX Imaging of the Bimodal Sphere Sample ...................... S-13
SI-14 – Approximate Calculation of NC Volumes During NC Growth............................. S-14
SI-15 – HAADF-STEM Images of the Irregular Yb:YLF Crystals ................................... S-15
SI-16 – EDX of the Irregular Yb:YLF Crystals.................................................................. S-16
SI-17 – Growth of Shuriken-Shaped LiF Microcrystals..................................................... S-17
SI-18 – Confirmation Shape LiF Cubes.............................................................................. S-18
SI-19 – Characteristics of Er:YLF Nanocrystals ................................................................ S-19

**SI-1 – Nucleation, Growth and Degradation of Yb:YLF Nanocrystals**

Figure S1 summarizes the most important steps of the nucleation, growth and degradation of Yb:YLF nanocrystals (NCs). At 300 °C, Yb:YLF spheres nucleate as a result of the sudden release of fluoride ions from the thermal decomposition of the TFA-based precursors (Figure S1a). At 330 °C, shown in Figure S1b, a bimodal distribution of nanospheres is observed. After 5 minutes (Figure S1c), a mixture of amorphous Yb:YLF nanospheres and crystalline Yb:YLF bipyramidal NCs are found. Generally, after 25 minutes, the synthesis is stopped and the product is recovered (Figure S1d). If the synthesis is not stopped at 25 minutes, but kept at 330 °C for 24 hours, the Yb:YLF NCs disintegrated, resulting in the presence of recrystallized microcrystalline LiF cubes (Figure S1e), and remaining Yb:YLF crystals (Figure S1f).

Figures S1a–c were obtained from a different synthesis batch, showing the reproducibility of the formation and transformation of the Yb:YLF spheres.

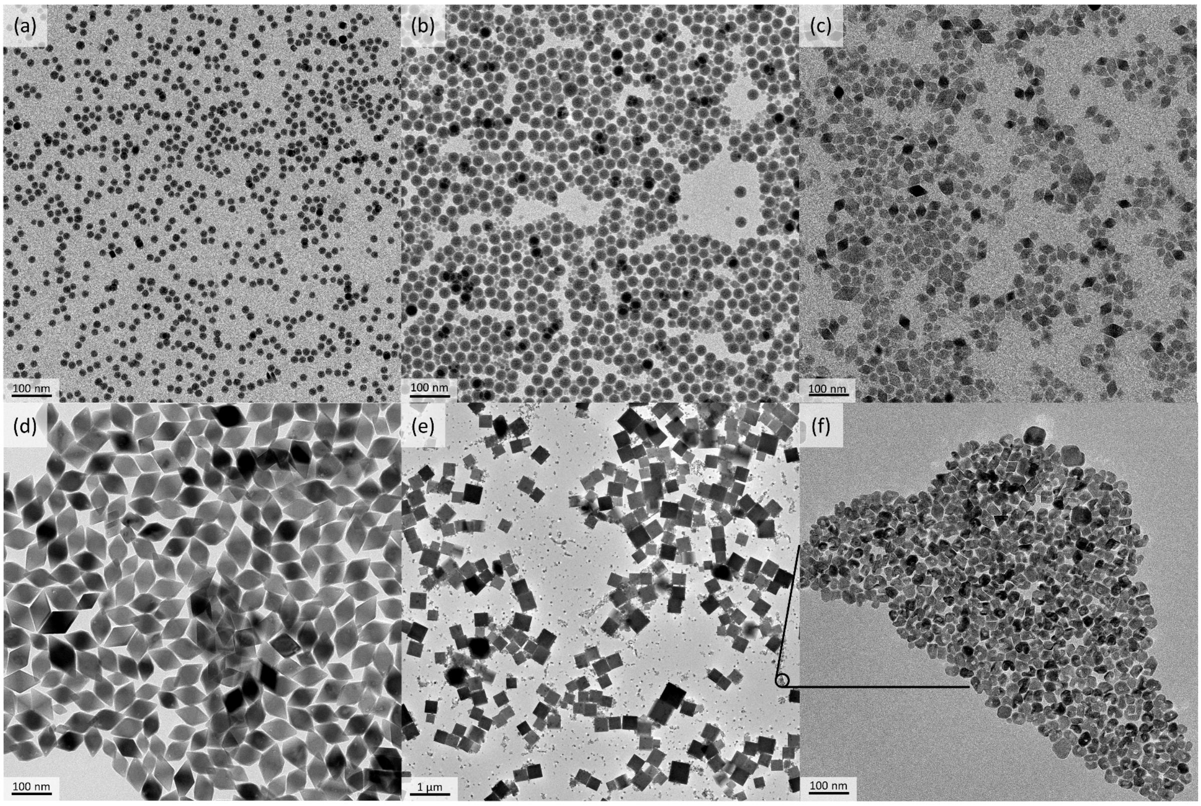


Figure S1. (a) Yb:YLF spheres obtained by taking an aliquot during the synthesis at 300 °C and (b) 330°C during heating up. (c) After 5 minutes at 330 °C, bipyramidal, crystalline Yb:YLF NCs were found among the Yb:YLF nanospheres. (d) NCs obtained after 25 minutes at 330 °C show the intended end product. (e) If the NCs are kept at the synthesis conditions for 24 hours, large cubes and (f) small irregular crystals are recovered. The image shows that no bipyramidal Yb:YLF NCs were present, indicating the observed particles are a degradation product of the Yb:YLF NCs.

## SI-2 – HAADF-STEM and EDX Images of Yb:YLF Spheres

In addition to Figure 2 in the main text, Figure S2 shows larger-area HAADF-STEM and EDX images of the Yb:YLF nanospheres obtained at 300 °C.

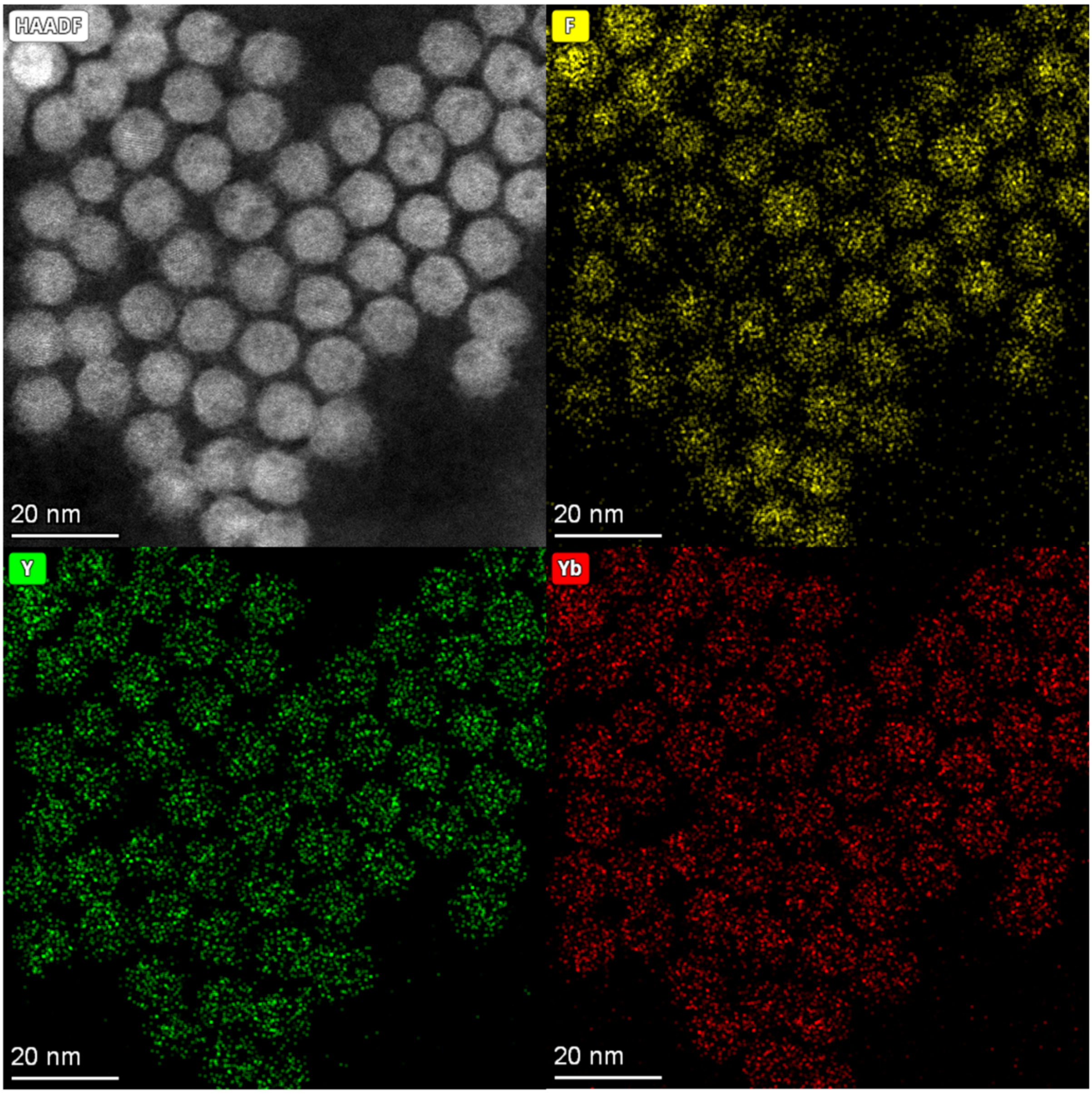


Figure S2. HAADF-STEM images and EDX elemental maps of the nanospheres obtained at 300 °C, during the heating up stage. This image shows the presence of fluoride, yttrium and ytterbium in the spheres.

## SI-3 – Yttrium and Ytterbium Distribution in Yb:YLF Spheres

For the nanosphere sample shown in Figure 2 of the main text, the yttrium and ytterbium distribution was determined using EDX. As can be seen in Figure S3, little to no signal of Y and Yb is outside of the spheres and a very similar signal ratio is found for Y:Yb in the nanospheres.

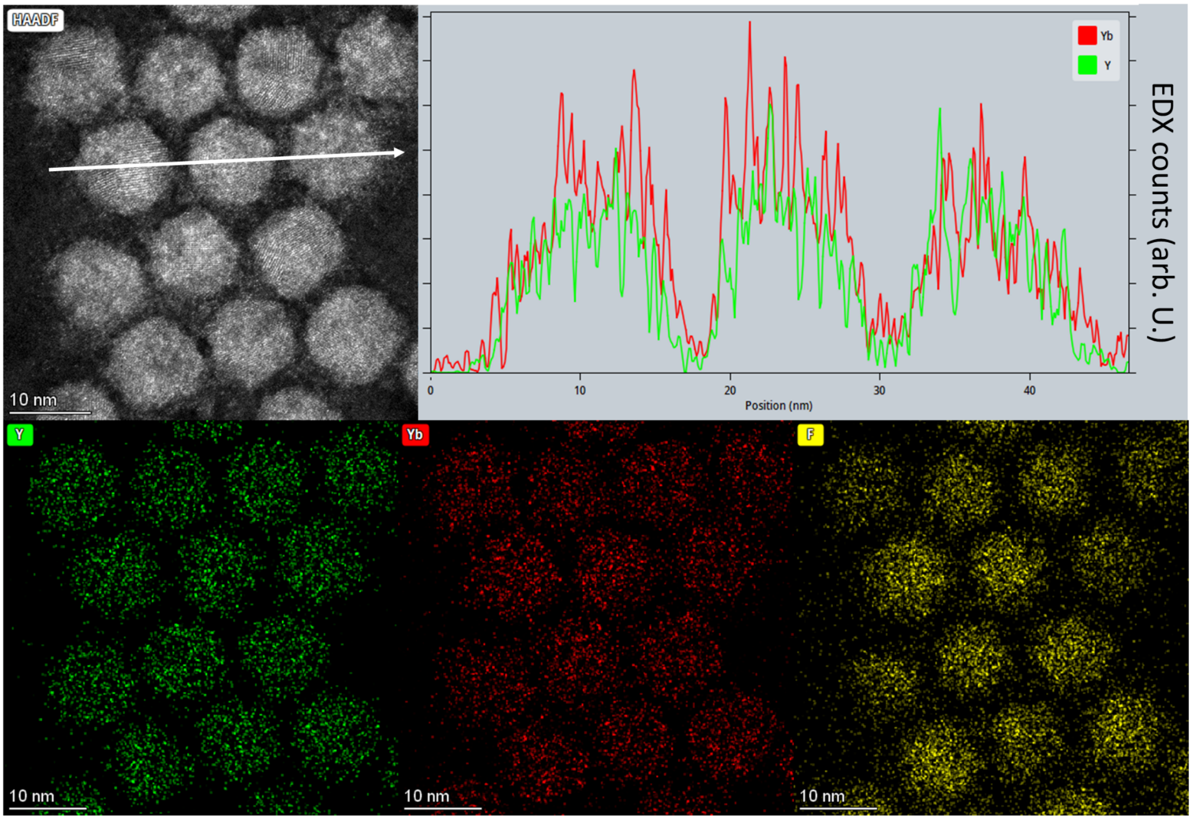


Figure S3. An EDX map of the nanospheres shown in Figure 2, performed in the direction of the arrow shown in the HAADF-STEM image, shows that the Y:Yb ratio is consistent in the nanospheres.

## SI-4 – XRD References for LiF, $YF_3$ and $LiYF_4$

Figure S4 shows the crystallographic information obtained using XRD from research grade LiF (99.995%) from Sigma Aldrich and $YF_3$ (99.9% from VWR). The XRD of $LiYF_4$ is obtained from synthesized NCs (without Yb-dopant). Important to notice is the large difference in the number and positions of the reflections between each material, significantly facilitating the differentiation between the materials. The values of a, b and c were calculated using the XRD diffractograms and the shown Miller indices were determined using the values for a, b and c. These values are used to determine the composition of the nanospheres, as shown in SI-10.

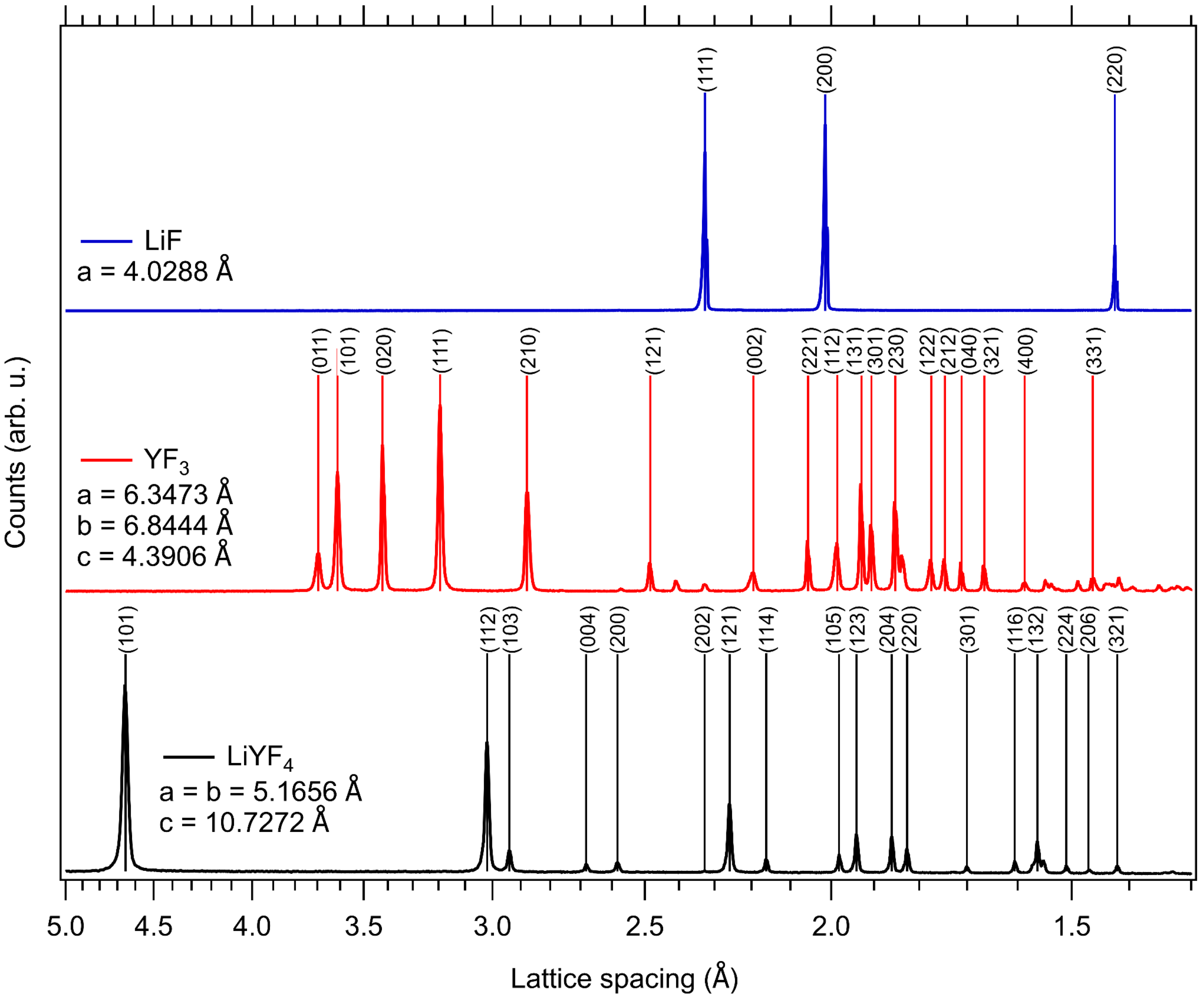


Figure S4. XRD diffractograms of LiF, $YF_3$ and undoped $LiYF_4$ with their calculated unit cell parameters and assigned reflections. All values are converted from 2 theta to lattice spacing in the unit cell, in order to obtain the values for a, b and c as indicated in the figure.

## SI-5 – Structural Analysis of Yb:YLF Spheres and Simulations of Unit Cell Reflections

Figure S5a shows the ED and XRD of the nanospheres, and the XRDs of the materials the nanospheres are most likely composed of. This figure shows that the nanospheres ED and XRD are most comparable with the XRD of $YF_3$. Although it is clear that the spheres are not composed of LiF (as additionally confirmed through EDX), unit cell reflections of $LiYF_4$ could possibly still result in the ED as shown in Figure S5b. To confirm that the reflections observed are not coming from $LiYF_4$, we simulated the expected reflections for $(1)^3$, $(1.5)^3$ and $(2)^3$ unit cells of $LiYF_4$ and $YF_3$, as shown in Figure S5c. This figure again shows that the experimental data is in best agreement with the simulated ED of $(1.5)^3$ to $(2)^3$ unit cells of $YF_3$. We therefore conclude that any signs of crystallinity in these spheres comes from local, short-range $YF_3$ order.

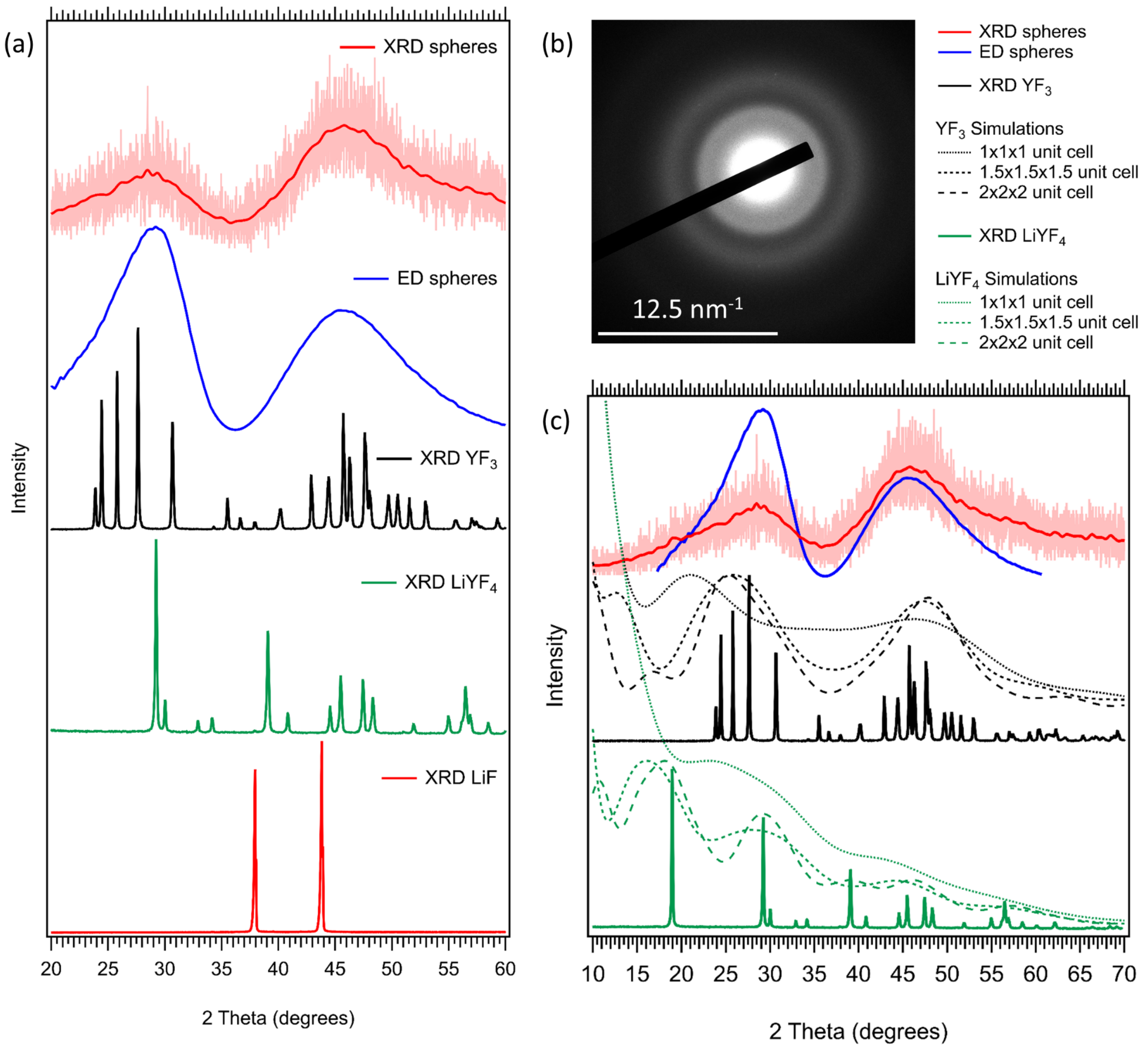


Figure S5. (a) XRD and ED diffractograms from the nanosphere sample, and XRD diffractograms of $YF_3$, $LiYF_4$ and LiF for reference. (b) ED from the nanospheres, of which the extracted pattern is shown in (a) and (c). (c) Simulations of the expected reflections of $YF_3$ and $LiYF_4$ for a 1x1x1, 1.5x1.5x1.5 and 2x2x2 unit cells.

## SI-6 – Nanocrystal Analysis Using Electron Diffraction

Figure S6 shows EDs of amorphous Yb:YLF nanospheres (top), undoped $LiYF_4$ NCs (middle) and LiF cubes (bottom). Comparing the XRD patterns from Figure S4 to the extracted diffraction patterns shows the accuracy of ED in this case. This figure shows that indeed the first sample of nanospheres is mostly amorphous due to the absence of sharp reflections.

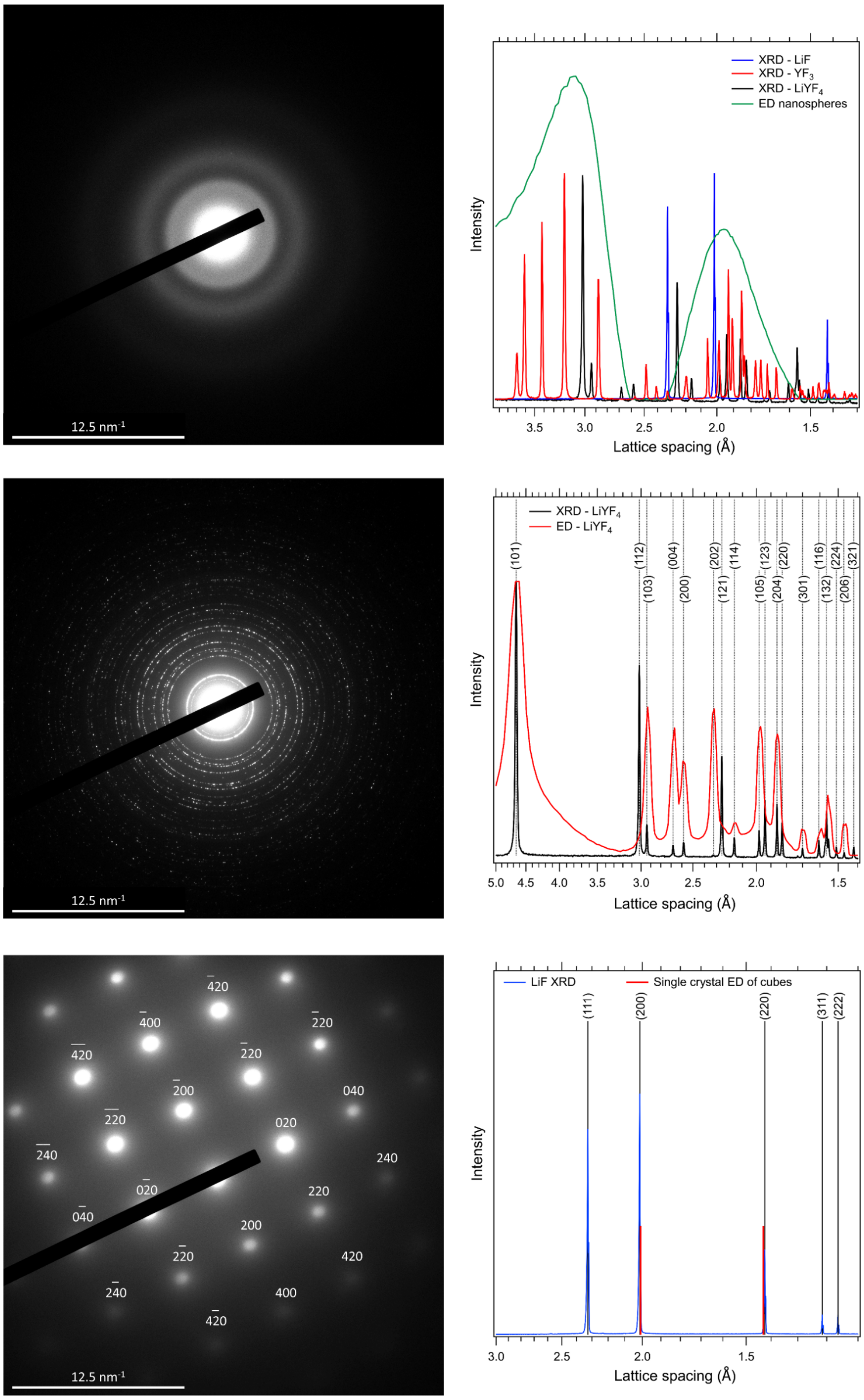


Figure S6. EDs and their extracted diffraction patterns from (top) the Yb:YLF nanospheres, (middle) $LiYF_4$ NCs and (bottom) LiF cubes. The first two EDs are small area EDs and hence show up as rings. The LiF ED is obtained from a single cube and hence shows a single crystal diffraction pattern.

## SI-7 – Optical Properties of Yb:YLF Nanospheres

Emission spectra were taken of both the Yb:YLF nanospheres and the bipyramidal NCs. Figure S7 shows the emission spectra for 25% ytterbium-doped particles. The emission spectrum of the spheres is similar to the emission of free $Yb^{3+}$ ions, indicating the spheres are not consisting of crystalline Yb:YLF. The emission of the bipyramidal NCs shows more emission features due to crystal field splitting, which is not observed in the spectrum of the spheres. The photoluminescence lifetime of the Yb-emission of the spheres (Figure S8) is much lower than usually seen for the parity forbidden transition (~2 ms), indicating that most of the excitations are lost to non-radiative processes.

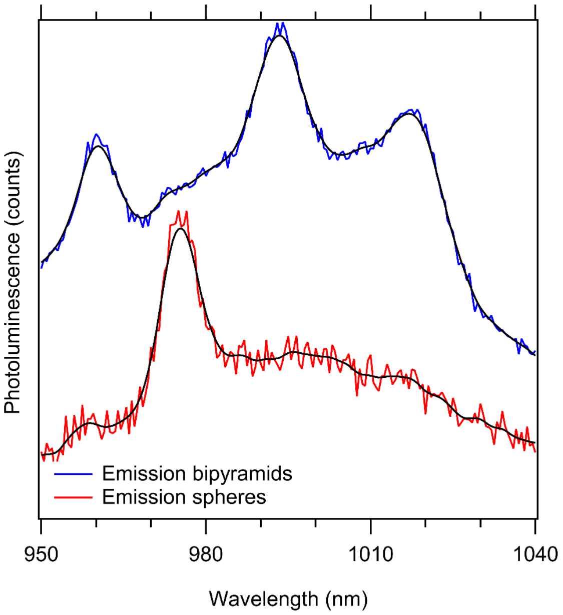


Figure S7. Emission spectra of 25% doped Yb:YLF nanospheres isolated at 300 °C, and 25% doped Yb:YLF bipyramidal NCs isolated after 25 minutes at 330 °C. $\lambda_{ex}$ = 930 nm for both samples.

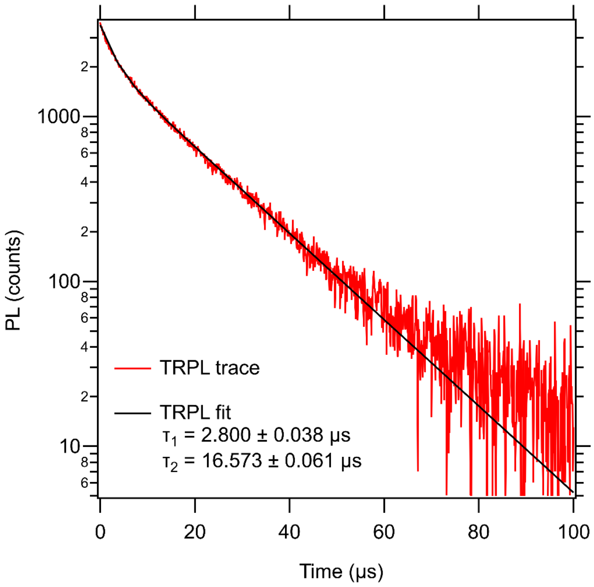


Figure S8. Photoluminescence decay of 25% doped Yb:YLF nanospheres of Figure S7. $\lambda_{ex}$ = 930 nm and $\lambda_{em}$ = 995 nm.

## SI-8 – Analysis of Nanosphere Material Composition Through e-Beam Crystallization

Because lithium is too light to be imaged with EDX, and because the spheres are initially amorphous, analyzing the composition of the spheres is not straightforward. The material can either be Yb:$YF_3$, or Yb:YLF. However, the amorphous spheres showed an increase of crystallinity when exposed to the electron beam during the HAADF-STEM measurements. From the arrangement of the yttrium ions in the crystallized spheres, we can already get more information to differentiate between Yb:$YF_3$ and Yb:YLF, as explained in SI-9.

From Figure S9, it is clear that the yttrium ions arrange into a hexagonal lattice. This structure is identical to that observed in Figure S10d-f. Therefore, it indicates that the spheres consist of Yb:YLF, and not of Yb:$YF_3$. However, more proof is needed.

The increase of crystallinity furthermore allows to extract a lattice parameter from the nanospheres, allowing to distinguish the material from its crystal structure, as is shown in SI-10.

Performing small area ED on these crystallized spheres remains practically impossible, due to the limited crystallized area, as well as the overall stability of the nanospheres. In Figure S9, the transformation from amorphous spheres at the 1$^{st}$ frame to the spheres showing crystallized areas in the 12$^{th}$ frame is clear. However, at the 100$^{th}$ frame, apart from crystallized areas, clear beam damage has affected the nanospheres.

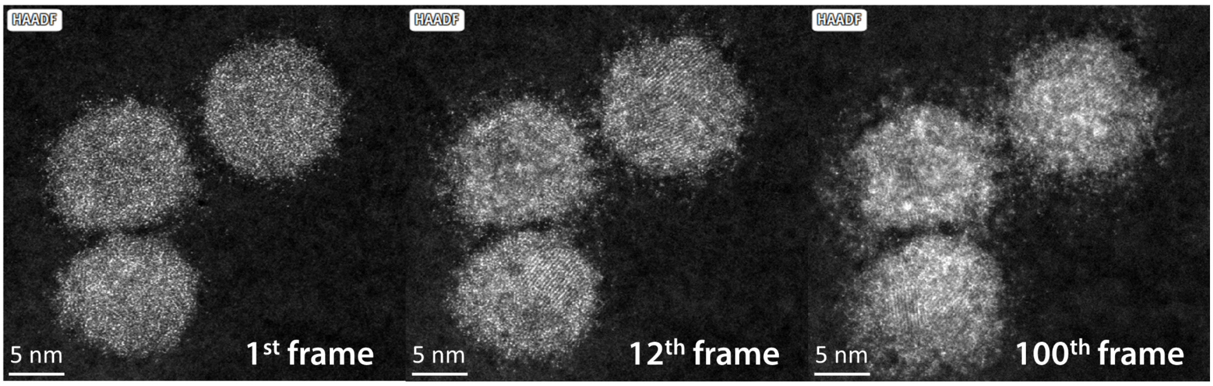


Figure S9. HAADF-STEM images of 3 Yb:YLF nanospheres, exposed to the electron beam for different amounts of time (*i.e.* measurement frames). The images show the 1$^{st}$, 12$^{th}$ and 100$^{th}$ frame taken. The 1$^{st}$ frame shows fully amorphous spheres with no signs of crystallinity. The 12$^{th}$ frame however shows clear presence of crystal lattices. The particles are however not stable under these conditions, as the 100$^{th}$ frame shows much more degradation of the spheres.

## SI-9 – Using the Location of Yttrium Ions to Distinguish Between $YF_3$ and $LiYF_4$

For samples where too little crystalline material is present, analyzing the material's composition through XRD and ED becomes impossible. However, as long as a small amount of material is crystalline (or crystallizes), a proper idea of the bulk composition can be obtained. In the case shown in Figure S10, only the yttrium ions are imaged (as the fluoride and lithium ions are in comparison too light).

In the top part of Figure S10, we show the arrangement of yttrium ions in $YF_3$ nanowires, found as a minor side product in one of the syntheses performed. The bottom part of Figure S10 shows yttrium ions in $LiYF_4$, obtained from the irregular Yb:YLF crystals (Figure S17), which were imaged through both HAAFD-STEM (right) and integrated differential phase contrast measurements (iDPC, middle). This figure clearly shows that the yttrium ions in $YF_3$ are found in pairs. The yttrium ions in $LiYF_4$ form a continuous and evenly spaced network throughout the lattice.

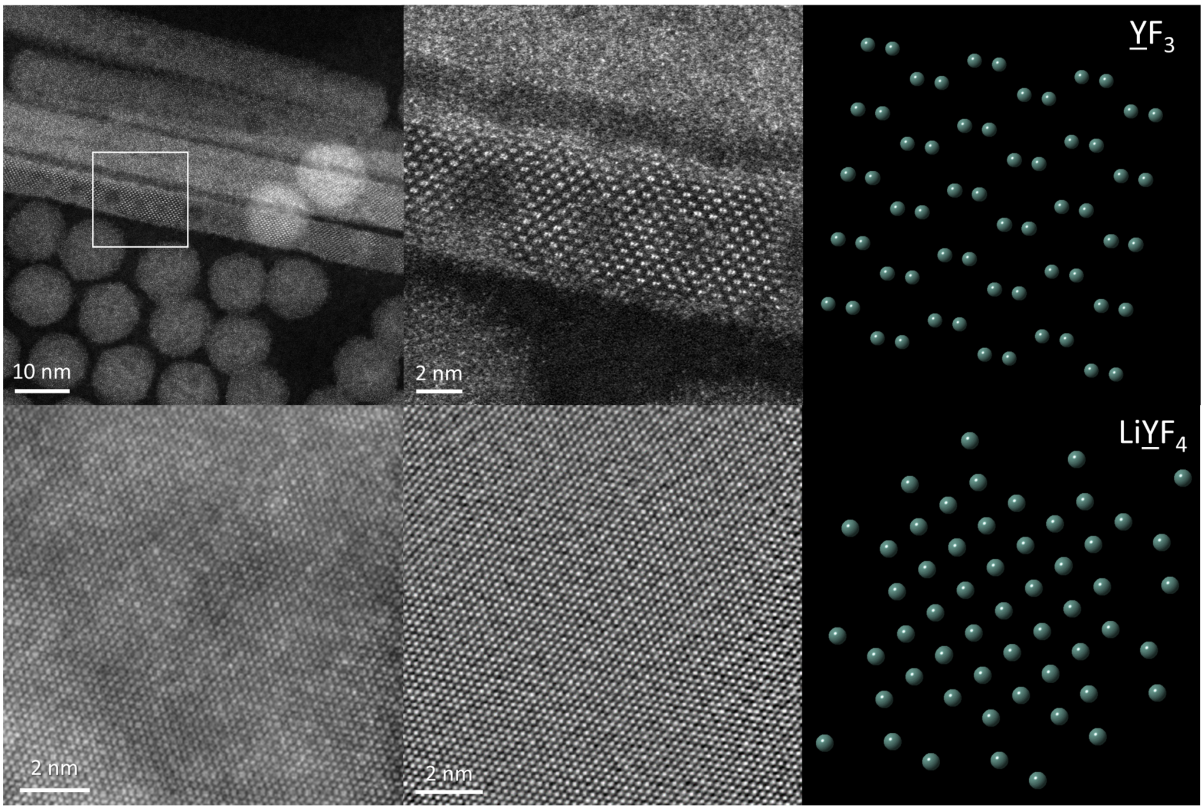


Figure S10. (top) HAADF-STEM images of $YF_3$ nanowires, found as a minor side product in one of the syntheses performed. When zooming in to an atomic level, it can be seen that the yttrium ions are paired, as shown in the top left. (bottom) For $LiYF_4$, the location of the yttrium ions is very different. Both by HAADF-STEM and iDPC imaging, it can be seen that the yttrium ions are unpaired and reside in a continuous hexagonal network.

## SI-10 – Analysis of Nanosphere Composition Using its Lattice Parameter

From the, under the e-beam crystallized, nanospheres, the $d_{hkl}$ value was determined from the observed atomic lattices. In this case, the HAADF-STEM image shown in the main text was used. Due to the lack of contrast, and the possibility of slight misalignments, this analysis is not error-free.

To reduce the error, the value for $d_{hkl}$ was averaged over 11 lattices, as shown in Figure S11. The $d_{hkl}$ value determined equals 3.03(6) Å. By using the XRDs of $YF_3$ and $LiYF_4$ (SI-4), it was concluded that this value corresponds to the (112) plane of $LiYF_4$, which has a $d_{hkl}$ value of 3.02 Å as shown in Figure S11.

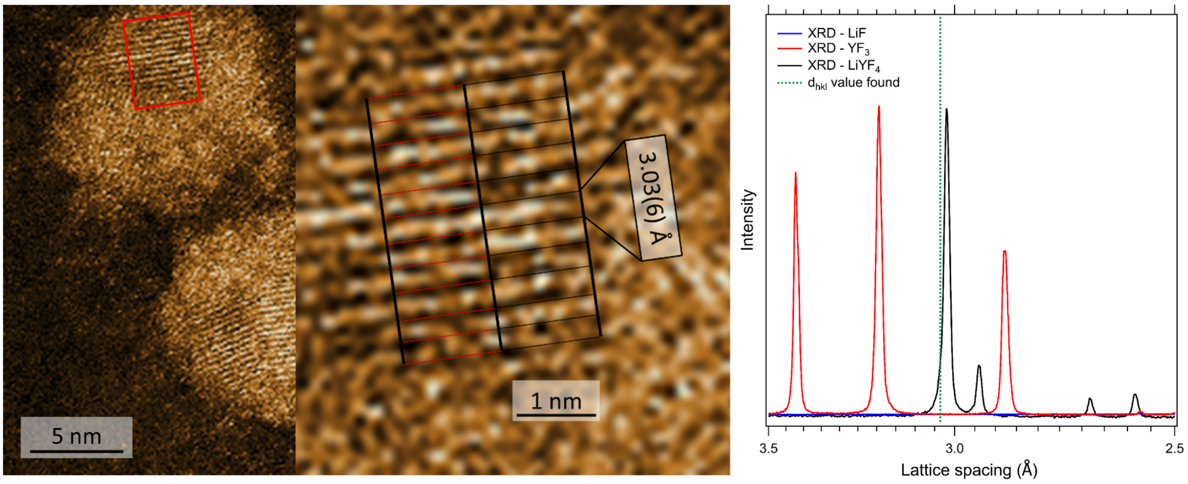


Figure S11. An analysis of the lattice parameter of the partially crystallized nanospheres. The image used is the same as shown in Figure 2 of the main text, but with enhanced contrast. The obtained $d_{hkl}$ value is 3.03(6) Å, which corresponds to the (112)-reflection of $LiYF_4$.

## SI-11 – XPS Measurements of Yb:YLF Nanospheres

XPS measurements were performed to measure if lithium ions are present in the nanospheres and, if so, in what ratio it is found compared to the lanthanide and fluoride ions. As shown in Figure S12a, lithium is clearly measured, and in roughly a 1:1.5 ratio of lithium:lanthanide, which of the expected compounds resembles Yb:YLF best. This is a strong indication that the analyses shown in SI-9 and SI-10 correctly identify the nanospheres as Yb:YLF.

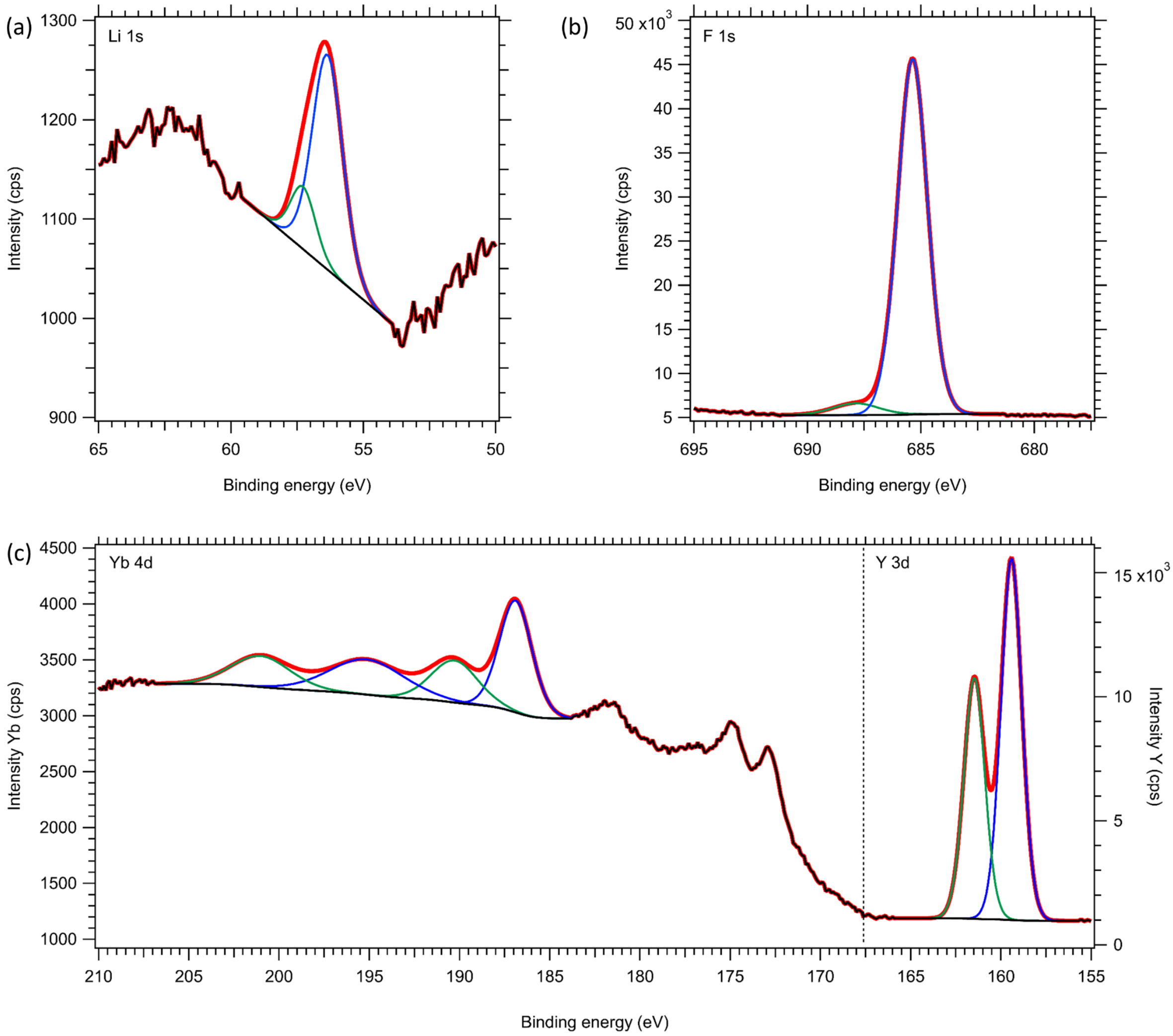


Figure S12. XPS spectra at element-specific binding energies, showcasing the presence of (a) Li, (b) F and (c) Y and Yb. Especially the presence of Li, which can hardly be measured using other techniques, aids in proving that the spheres consist of Yb:YLF, and not of Yb:$YF_3$.

## SI-12 – XRD on Thermally Annealed Nanospheres

In order to fully analyze the composition of the nanospheres, the sample (that was washed thrice to remove any residual non-incorporated ions) was thermally annealed at 330 °C for 25 minutes (mimicking the NC synthesis) under inert atmosphere. The material was then analyzed using XRD. As shown in Figure S13, the XRD pattern after annealing shows strong similarities with that of $LiYF_4$, with a minor $YF_3$ phase present (in agreement with the XPS results shown in SI-11). This proves that lithium is present in the nanospheres, and that the material thus mostly consists of amorphous Yb:YLF. A slight shift of the corresponding angles is observed for the thermally annealed nanospheres compared to pure $LiYF_4$, which is likely a result of a slight lattice contraction due to the presence of 25% $Yb^{3+}$.

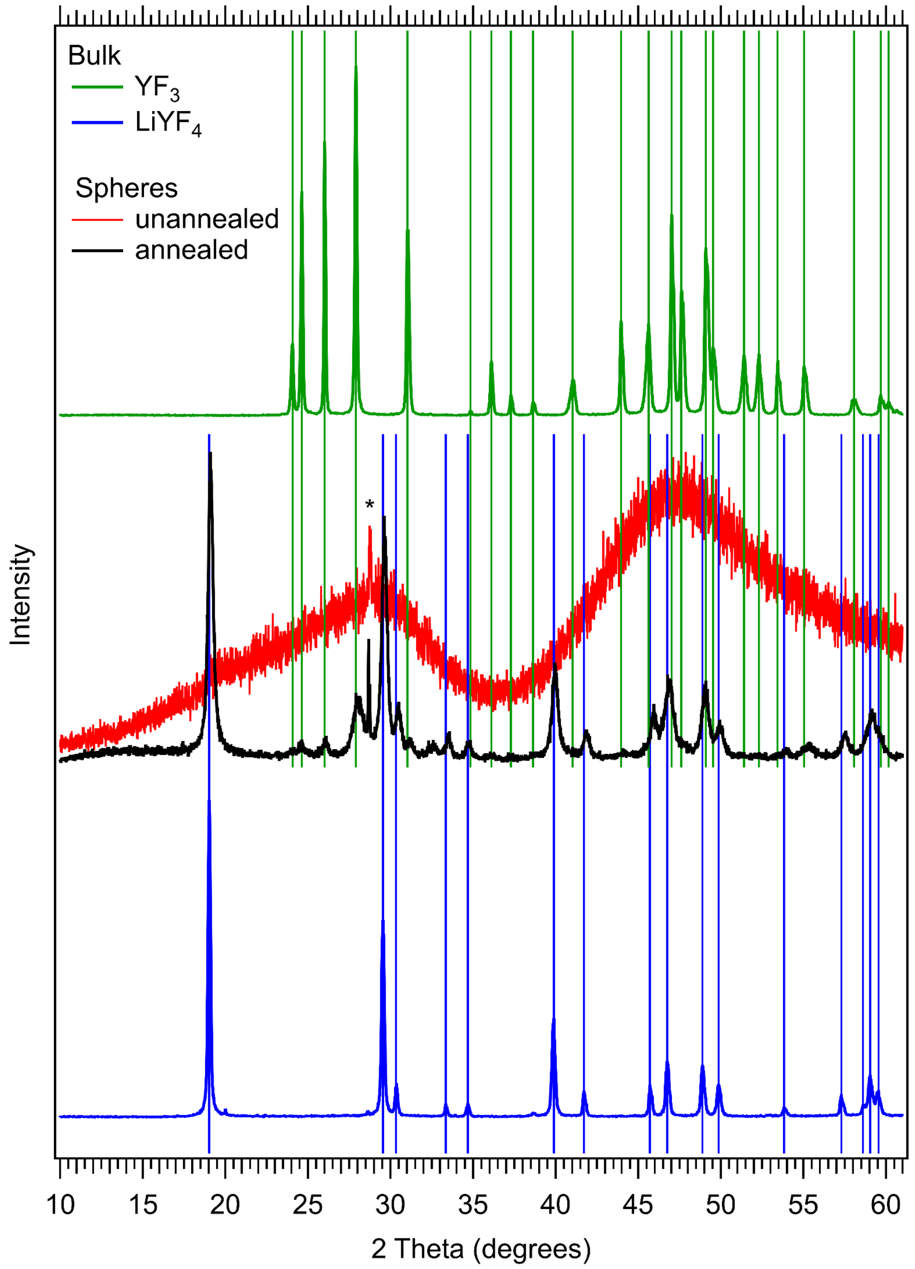


Figure S13. XRD diffractograms of $YF_3$ (green), $LiYF_4$ (blue) and the nanospheres before (red) and after (black) thermal annealing. The nanosphere-diffractogram after annealing shows strong similarities with $LiYF_4$. The sharp reflection at 28.7 degrees (*) corresponds to the (111) reflection of the silicon substrate that was used.

## SI-13 – HAADF-STEM and EDX Imaging of the Bimodal Sphere Sample

Figure S14 shows two HAADF-STEM images and corresponding EDX mapping of the bimodal sphere sample. From these images, no difference is observed between the large and small spheres. As in the main text we conclude that $YF_3$ alone is too soluble, it was concluded that these spheres are both Yb:YLF, undergoing Ostwald ripening as shown in Figure S15.

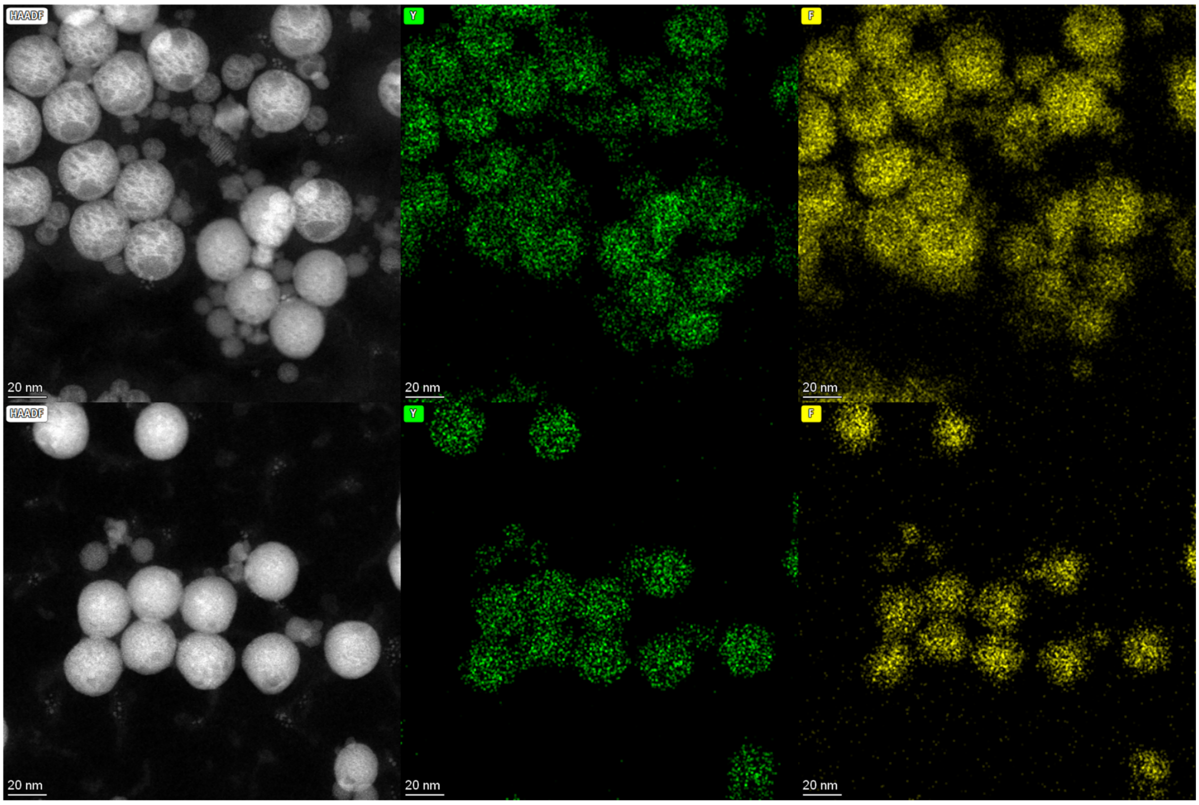


Figure S14. HAADF-STEM images and EDX elemental maps of 2 sets of bimodal spheres. Composition-wise, these spheres are concluded to be no different than the spheres observed and isolated at 300 °C.

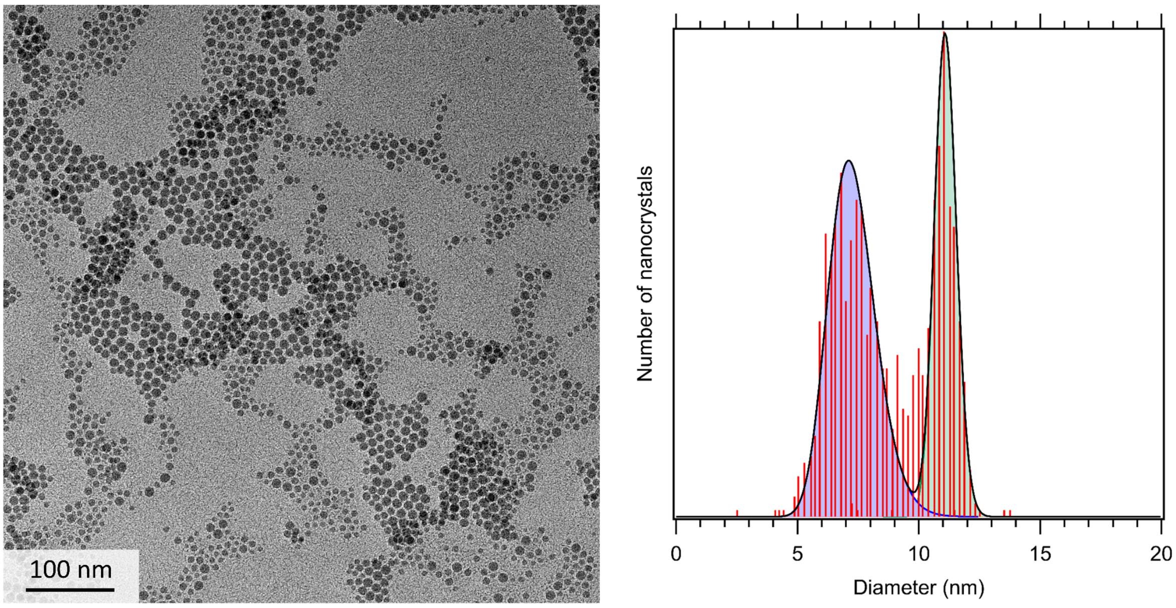


Figure S15. Larger view of the TEM image displayed in Figure 1c, and the obtained size distribution shown in Figure 3c showcasing the Ostwald ripening stage of the Yb:YLF spheres.

## SI-14 – Approximate Calculation of NC Volumes During NC Growth

In order to understand whether or not the Yb:YLF spheres crystallized into bipyramidal NCs, or that they nucleate on longer times scales only, the approximate volumes of both NC types were calculated. From Figure S16, where we selected a large sphere and a small bipyramidal NC, it can be seen that the volumes are relatively similar, although slightly larger for the chosen spherical NCs.

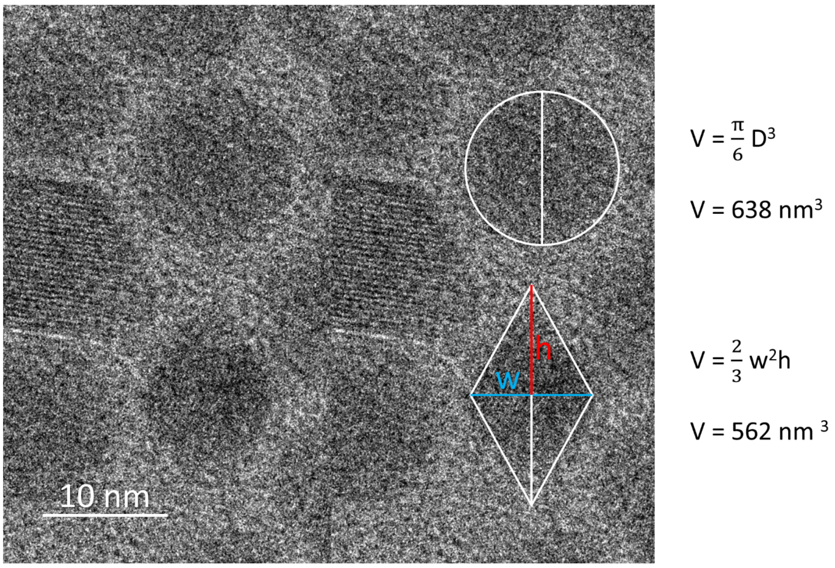


Figure S16. TEM image used for calculating the NC volumes. As can be seen, the volumes of the spherical NCs and the bipyramidal NCs do not differ much.

## SI-15 – HAADF-STEM Images of the Irregular Yb:YLF Crystals

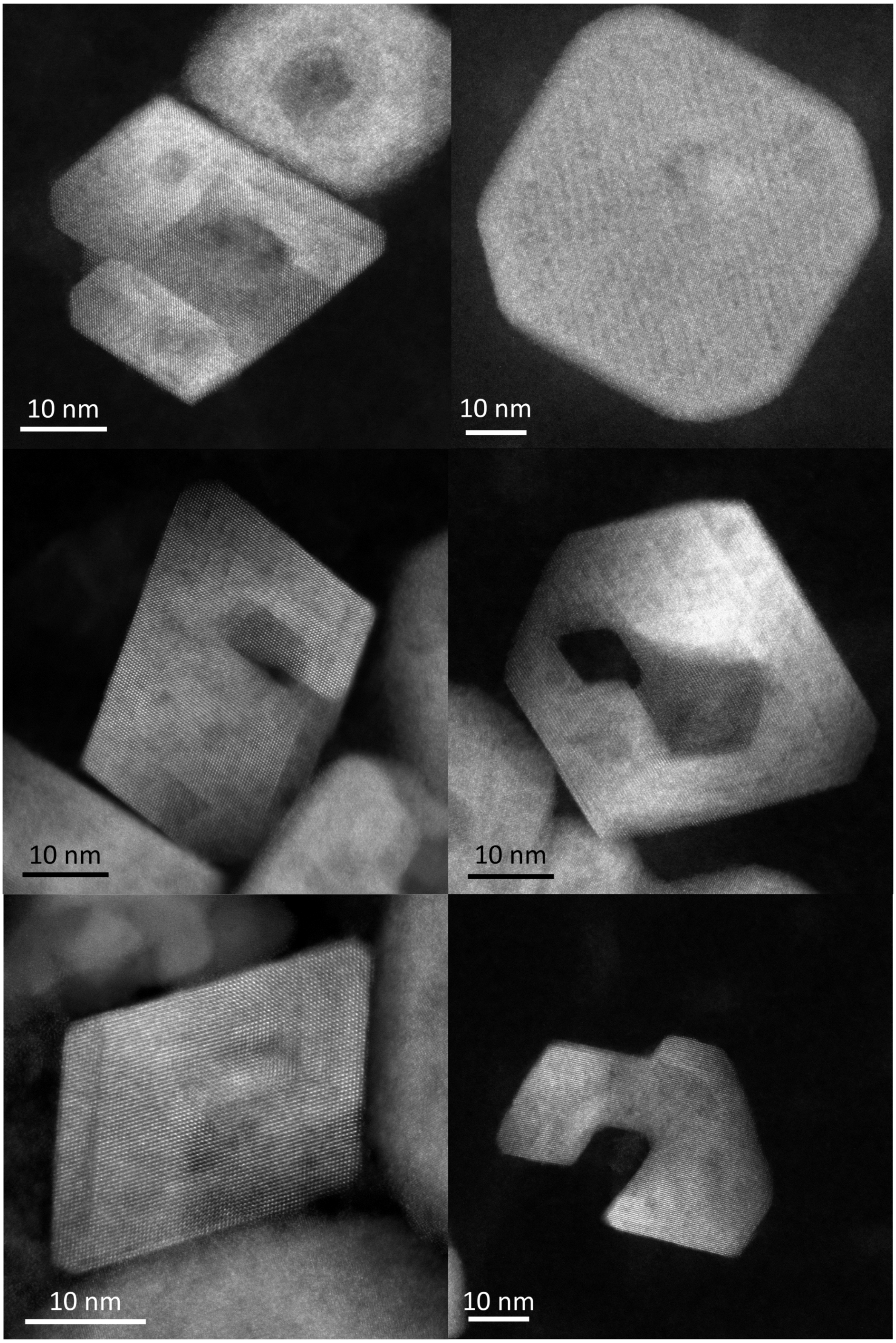


Figure S17. HAADF-STEM images of the irregular crystals. These images show the large variety in shapes, the large surface area and the high crystallinity for each particle. From the location of the yttrium ions (SI-9), it was concluded that these NCs consist of Yb:YLF.

**SI-16 – EDX of the Irregular Yb:YLF Crystals**

EDX elemental mapping was performed on certain crystals, as shown below. All particles showed the presence of fluoride, yttrium and ytterbium.

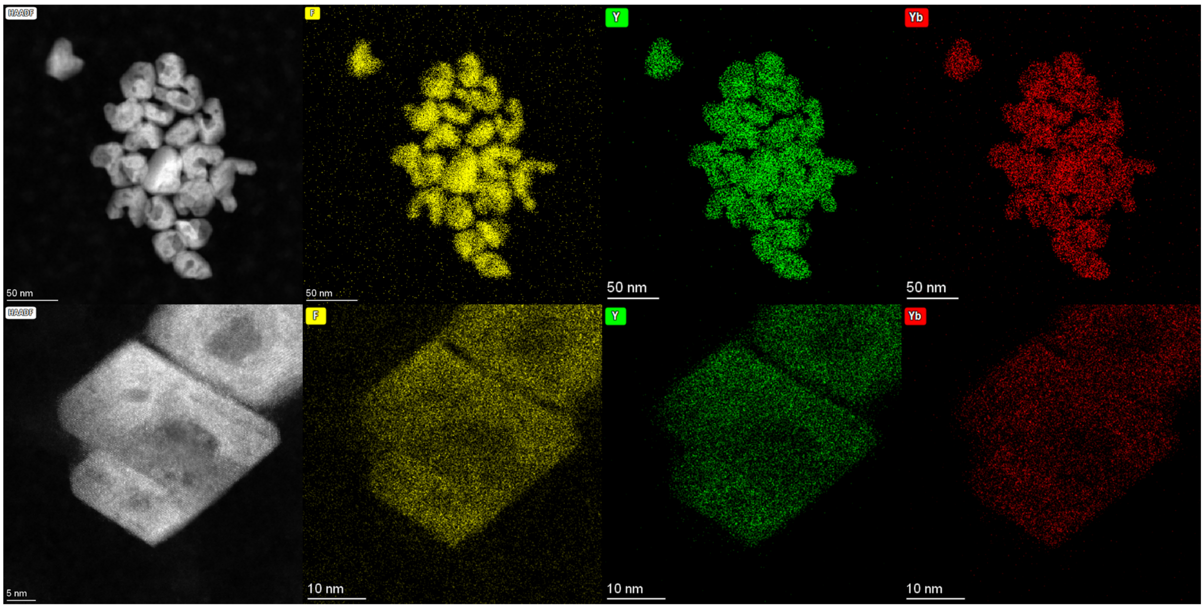


Figure S18. HAADF-STEM images and EDX maps of fluoride, yttrium and ytterbium showing that all the irregularly shaped crystals recovered after 24 hours consist of Yb:YLF.

## SI-17 – Growth of Shuriken-Shaped LiF Microcrystals

In order to understand how LiF cubes were recovered after 24 hours, a synthesis similar to the Yb:YLF NC synthesis was performed, but using solely LiTFA in order to form LiF. No particles were found at 270 °C, however at 285 °C, 300 °C, 315 °C, and 330 °C large shuriken-shaped LiF crystals were found. These crystals and their growth are shown in Figure S19. It is clear that these crystals are in a thermodynamically unfavorable state, due to the large surface area. When irradiating the crystals with an intense electron beam they burst, releasing a large number of smaller, cubic and highly crystalline LiF fragments (as shown in Figure 4f of the main text). It is important to note that the formation of Yb:YLF NCs is thus a symbiotic phenomenon between the cracking of LiTFA and $Y(TFA)_3$, preventing these LiF crystals from nucleating.

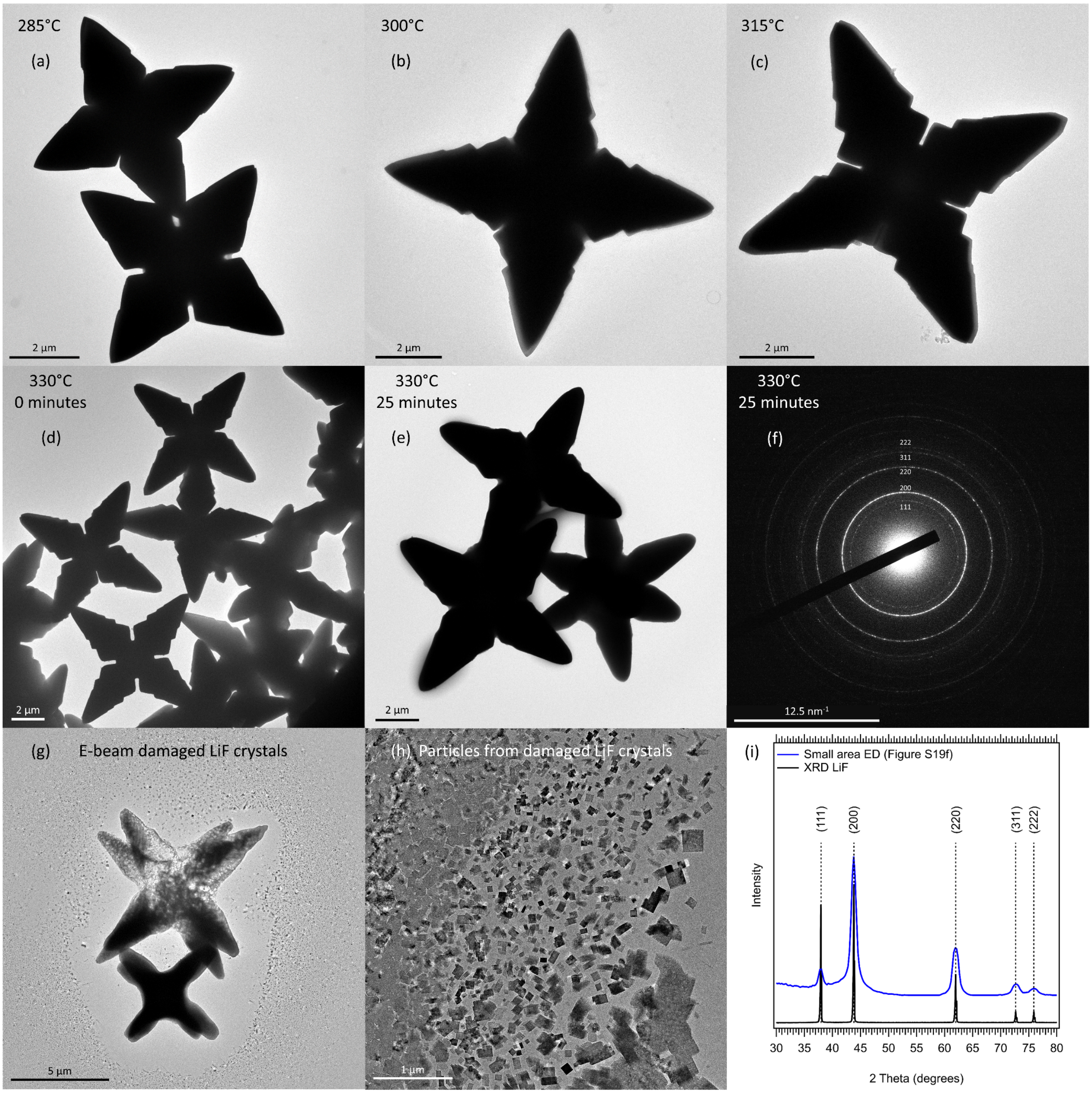


Figure S19. (a – e) Growth stages of shuriken-shaped LiF crystals synthesized by using solely LiTFA in the YLF synthesis. From 285 °C large crystals are found, which are unstable under the electron beam of the TEM and, as shown in (g – h) "explode" into small fragments. (i) ED on the fragments (f) of the damaged crystals confirms the material synthesized is LiF.

## SI-18 – Confirmation Shape LiF Cubes

Due to the limited electron density in LiF, TEM images alone could not confirm the dimensions of the LiF cubes that were found. To confirm that the material is indeed cube-shaped, tomographic measurements were performed in the TEM (JEOL JEM1400). As shown in Figure S20, the LiF material is indeed a near-perfect cube, and not (for example) a platelet.

Side view

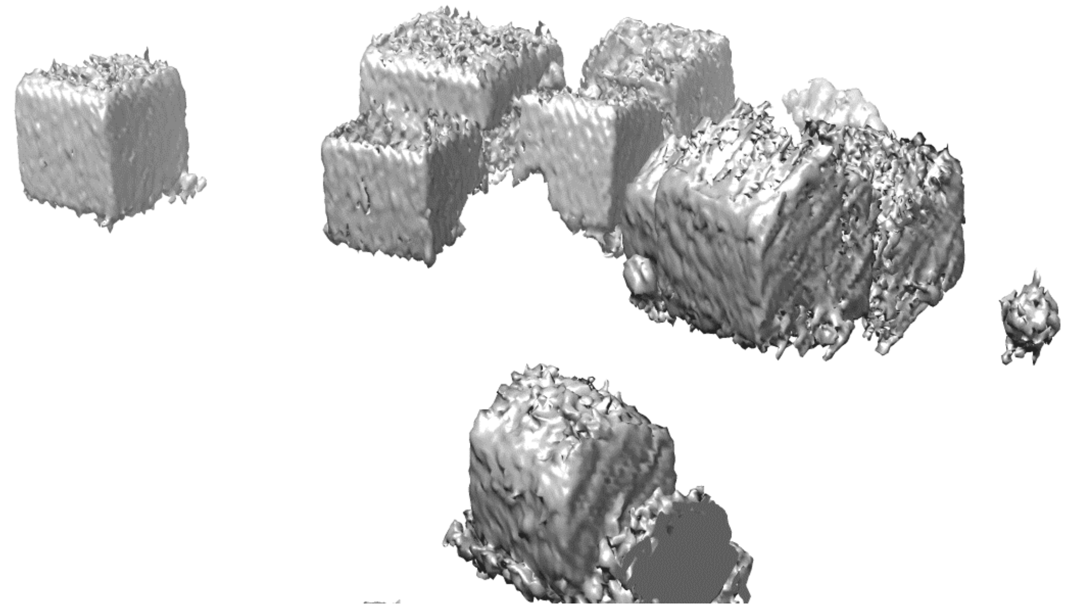

Front view

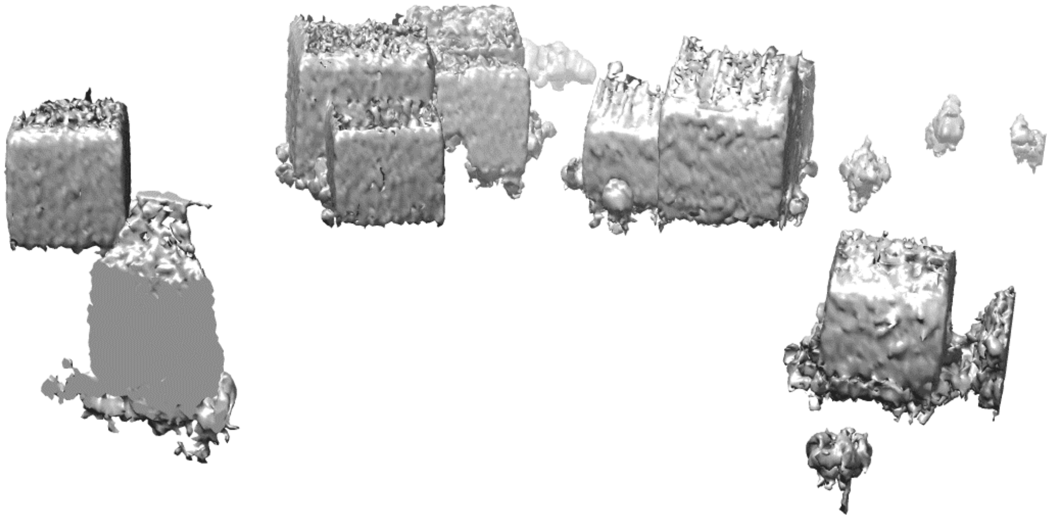

Figure S20. Side and front views of the LiF cubes that were obtained after 24 hours, showcasing that the material is cube shaped.

## SI-19 – Characteristics of Er:YLF Nanocrystals

The aim of this work is to show the nucleation and growth of $LiYF_4$ NCs, and the ease with which this host material can be doped with lanthanide ions. Our study focuses on Yb:YLF, as $Yb^{3+}$ is most often incorporated in $LiYF_4$ NCs, either as sole dopant or as activator ion for other lanthanides. This work, however, does not pertain solely to Yb:YLF but, due to the similarities in chemical nature, can be applied for any lanthanide-doped $LiYF_4$ NC synthesis. To show this, we synthesized 30% erbium-doped Er:YLF NCs. The TEM images (Figure S21a) show a similar size and shape of the NCs in the sample. EDX measurements (Figure S21b) show that the doping density corresponds to 31.9% (slightly overestimated due to a slight background signal), hence analogous to $Yb^{3+}$, $Er^{3+}$ incorporates as expected from the feed fraction. ED (Figures S21c and S21d) shows that the crystals have a phase-pure scheelite crystal structure, with a small lattice contraction compared to pure $LiYF_4$, due to the incorporation of slightly smaller $Er^{3+}$ ions. The PL spectrum, Figure S21e, confirms the incorporation of $Er^{3+}$ ions as the spectrum is similar to that of bulk Er:YLF, both for the visible (VIS) and the near-infrared (NIR) spectrum.

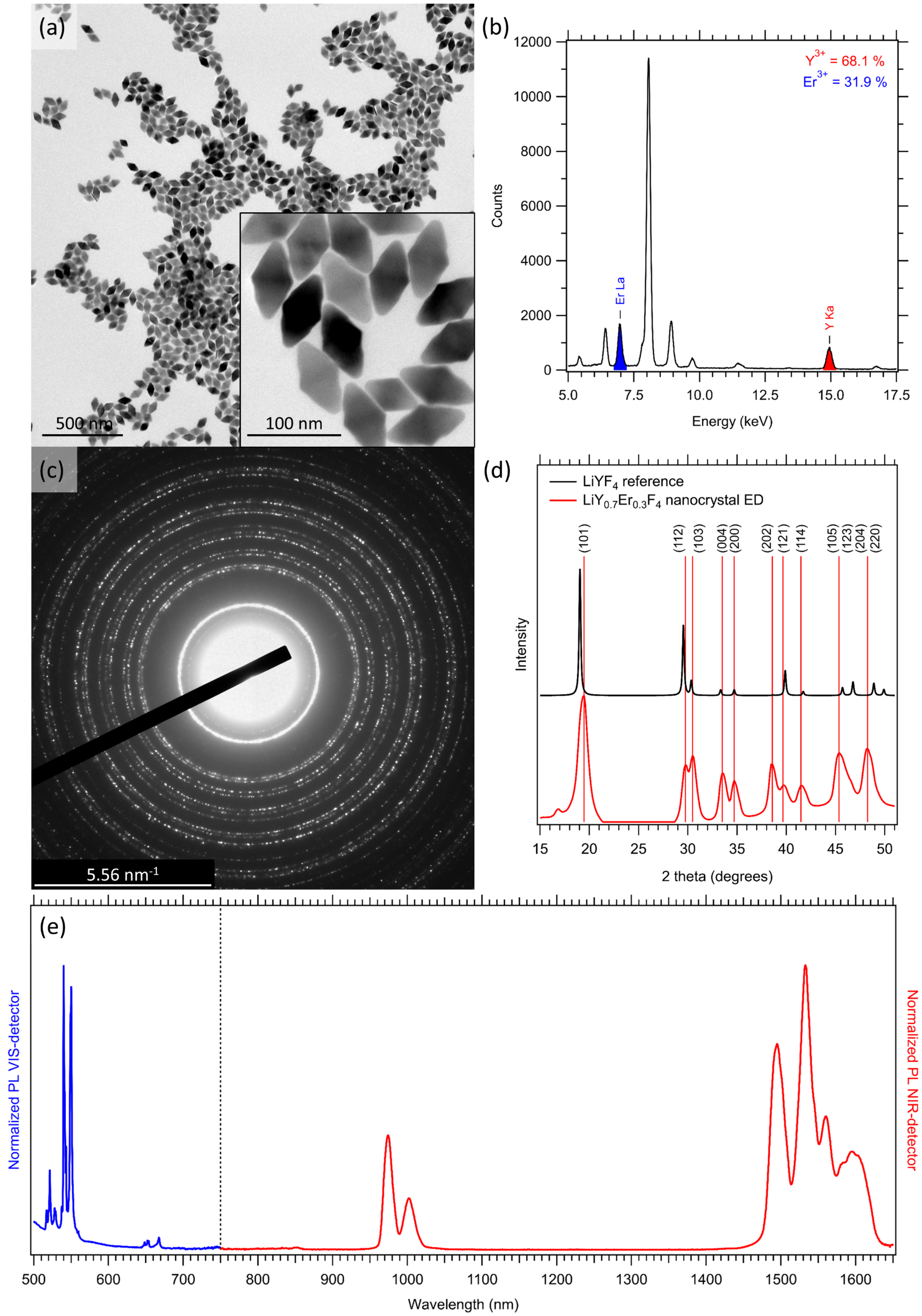


Figure S21. (a) TEM images, (b) EDX, (c, d) ED and (e) PL of an Er:YLF NC sample with an $Er^{3+}$-doping of 30%. All observations agree with the expectation that this work does not only pertain to Yb:YLF NC syntheses, but is also true to other Ln:YLF NC syntheses.
